# Thioguanine-based DENV-2 NS2B/NS3 protease inhibitors: Virtual screening, synthesis, biological evaluation and molecular modelling

**DOI:** 10.1371/journal.pone.0210869

**Published:** 2019-01-24

**Authors:** Maywan Hariono, Sy Bing Choi, Ros Fatihah Roslim, Mohamed Sufian Nawi, Mei Lan Tan, Ezatul Ezleen Kamarulzaman, Nornisah Mohamed, Rohana Yusof, Shatrah Othman, Noorsaadah Abd Rahman, Rozana Othman, Habibah A. Wahab

**Affiliations:** 1 School of Pharmaceutical Sciences, Universiti Sains Malaysia, Minden, Pulau Pinang, Malaysia; 2 Faculty of Pharmacy, Sanata Dharma University, Maguwoharjo, Sleman, Yogyakarta, Indonesia; 3 School of Data Sciences, Perdana University, Blok B and d1, MARDI Complex, Jalan MAEPS Perdana, Serdang, Selangor; 4 Department of Pharmaceutical Chemistry, Kulliyah of Pharmacy, International Islamic University Malaysia, Kuantan, Pahang, Malaysia; 5 Advanced Medical and Dental Institute, Universiti Sains Malaysia, Bertam, Pulau Pinang, Malaysia; 6 Department of Molecular Medicine, Faculty of Medicine, Universiti Malaya, Kuala Lumpur, Malaysia; 7 Department of Chemistry, Faculty of Science, Universiti Malaya, Kuala Lumpur, Malaysia; 8 Department of Pharmacy, Faculty of Medicine, Universiti Malaya, Kuala Lumpur, Malaysia; 9 Malaysian Institute of Pharmaceuticals and Nutraceuticals, Ministry of Science, Technology and Innovation, Halaman Bukit Gambir, Bayan Lepas, Pulau Pinang, Malaysia; UMR-S1134, INSERM, Université Paris Diderot, INTS, FRANCE

## Abstract

Dengue virus Type 2 (DENV-2) is predominant serotype causing major dengue epidemics. There are a number of studies carried out to find its effective antiviral, however to date, there is still no molecule either from peptide or small molecules released as a drug. The present study aims to identify small molecules inhibitor from National Cancer Institute database through virtual screening. One of the hits, **D0713** (IC_50_ = 62 μM) bearing thioguanine scaffold was derivatised into 21 compounds and evaluated for DENV-2 NS2B/NS3 protease inhibitory activity. Compounds **18** and **21** demonstrated the most potent activity with IC_50_ of 0.38 μM and 16 μM, respectively. Molecular dynamics and MM/PBSA free energy of binding calculation were conducted to study the interaction mechanism of these compounds with the protease. The free energy of binding of **18** calculated by MM/PBSA is -16.10 kcal/mol compared to the known inhibitor, panduratin A (-11.27 kcal/mol), which corroborates well with the experimental observation. Results from molecular dynamics simulations also showed that both **18** and **21** bind in the active site and stabilised by the formation of hydrogen bonds with Asn174.

## Introduction

Dengue, caused by Dengue Virus (DENV), is the most important mosquito-borne viral disease affecting the tropics and subtropics [1]. Endemic in more than 100 countries [2,3], the virus is estimated to cause 390 million infections each year [4]. DENV infections can result in serious diseases including dengue fever, dengue hemorrhagic fever (DHF), dengue shock syndrome (DSS) and even death [5]. There are no approved antiviral drugs for these diseases and currently, patients are treated with supportive care to relieve fever, pain, and dehydration [6]. A tetravalent dengue vaccine (CYD-TDENV or Dengvaxia), the first dengue vaccine has recently been registered in several countries [7]. Despite being a leading cause of hospitalisation and death among children in some Asian and Latin American countries [8], this vaccine is not recommended for use in children under 9 years of age due to safety concerns [7]. Therefore, there exists an urgent need for antiviral therapies to treat dengue.

DENV carries a positive single strand RNA in its genome and five serotypes (DENV-1 to 5) have been identified. DENV-2 is the most prevalent type in dengue epidemic, especially in the South East Asian region and has been associated with severe dengue cases [9]. The new serotype (DENV-5) [10] was discovered in 2013 in Sarawak, Malaysia further complicates the prevention and treatment of the disease. The virus genome is encoded by three structural proteins (C, prM, E) as well as seven non-structural proteins (NS1, NS2A, NS2B, NS3, NS4A, NS4B and NS5) [11]. Of these proteins, NS2B/NS3protease (NS2B/NS3pro) has been well studied and regarded as a promising target in anti-dengue discovery [12–15].

NS3 is a trypsin like serine protease which plays a role in post-translation in the virus maturation. This domain has a catalytic triad made up of His51, Asp75 and Ser135 and its activity is enhanced by NS2B as the cofactor [16,17]. This cofactor contributes to the NS3 activity through its hydrophilic region which is responsible for holding and promoting the activation of NS3 while the hydrophobic region takes part in membrane association upon the cleavage process [18,19].

Previous discoveries of dengue inhibitors by targeting NS2B/NS3pro activity were mainly based on the non-prime substrates which were identified by profiling dengue virus using tetrapeptides [20–22]. Significant challenges arise as the protein possesses a solvent-exposed, topologically shallow active site and dependent on the selectivity for substrates containing basic amino acids (arginine and lysine) at P1 and P2 positions [23]. Nevertheless, many peptidic or modified peptidic molecules have been discovered to have good NS2B/NS3pro inhibition activities [15, 24–30]. In addition, there are also reports of potent small molecule NS2B/NS3pro inhibitors from natural products (panduratin[31], agathisflavone and quercetin [32]), from synthetic medicinal chemistry (dehydronaphthalene [33], benzimidazole [34], and thiadiazoloacrylamide [35]) or from the utilisation of computational methods [36–39].

Here, we report the discovery of potential NS2B/NS3pro inhibitors designed based on thioguanine scaffold identified through the virtual screening of compounds library from National Cancer Institute (NCI) diversity set II. Twenty-four compounds were found as *in silico* hits based on the free energy of binding (ΔG_bind_) ranking from a total of 1990 compounds. Twenty of them were obtained from NCI for *in vitro* assay, out of which four demonstrated moderate inhibition towards DENV-2 NS2B/NS3pro (IC_50_ = 29–77 μM). Although Diversity0713 (**D0713**) is not the most potent compound identified in the virtual screening, the structure contains thioguanine (**TG** or 6-thioguanine) scaffold can be used as a template to develop a series of analogues as its simple chemical structure benefits feasible synthetic steps. **TG** showed 56% inhibition at 200 μg/mL (1.2 mM) indicating that even without any modification to the structure, the scaffold itself is able to inhibit the protease activity. This compound is a drug classed as anti-neoplastic agent and used with other compounds in treating leukemia [40] and has been investigated in many pharmacological activities such as immunomodulators in autoimmune diseases [41] and transplant graft rejection [42]. From this initial study, we are interested to utilise thioguanine scaffold in the design of DENV-2 NS2B/NS3pro inhibitor(s). Thus, in this study, we designed, synthesised thioguanine analogues and investigated their possible DENV-2 NS2B/NS3pro inhibition activity. We hope that this study could contribute to the efforts in discovering novel and potent anti-dengue agents.

## Materials and methods

### Virtual screening

Virtual screening was carried out using AutoDockVina [43] (www.autodock.scripps.edu). The DENV-2 NS2B/NS3pro model was taken from published article [44], where the model was built based on the DENV-2 complex cofactor-protease using the crystal structure of NS2B/NS3pro West Nile Virus (WNV) as the template. The docking procedure was initiated by the preparation of NS2B/NS3pro as a macromolecule using AutoDock Tools (version 1.5.6) with default parameters for docking with AutoDock Vina. The exhaustiveness was set to 8 and other parameters were unchanged. The centre of the grid box was set at 30.71, 50.48 and 4.10 Å in x, y, z coordinates, respectively, with a box size of 25 x 25 x 25 points. The internal validation was done by re-docking the tetrapeptide inhibitor (Bz-Nle-Lys-Arg-Arg-H) with the RMSD value not greater than 2Å. The external validation was done using panduratin A (a competitive inhibitor), that dock at the same binding site of tetrapeptide inhibitor. The *in silico* screening of 1990 ligands from NCI diversity set (II) was carried out using the docking parameters above. The hit compounds were ranked according to the free energy of binding (ΔG_bind_) and analysis of their binding modes were performed using Discovery Studio 3.5 (www.accelrys.com).

### DENV-2 NS2B/NS3pro expression and purification, optimum activity and inhibition assay

The DENV-2 NS2B/NS3pro expression was carried out according to the established method by Yusof *et al*., (2000) [45] with minor modifications according to published articles [46–50]. The plasmid encoding the NS2B/NS3 protease sequence from DENV-2 was transformed into *Escherichia coli* strain XL1-Blue transformed with pQE30.CF40.gly(T).NS3pro expression plasmid were grown in LB medium containing 10 μg/ml ampicillin at 37°C until the OD_600_ reached 0.6. First, the cells were incubated at 37°C, 200 rpm until OD_600_ reached ~0.6. One ml of isopropyl-β-D-thiogalactopyranoside (0.5 mM in LB medium) was added to the bacterial cells for 2 hours to induce protein expression. Expression of the recombinant protein was induced by the addition of 0.5 M IPTG and the culture was incubated for 2 hours.

The cells were harvested by centrifugation at 8000 rpm (Sorvall RC-5B Refrigerated Superspeed centrifuge) for 15 minutes at 80°C. The cell pellets were thawed (1 g) and resuspended in lysis buffer (5 mL) followed by mixing them using vortex until milky. For purification, cells were lysed by sonication conducting (6 times 15-second pulse, duty cycle 10%, output control no 3) using Ultrasonic Cell Disruptor, Branson Sonifier 450, Germany. The lysate was incubated on ice for 1 hour and then centrifuged at 8000 rpm for 1 hour at 4°C. The soluble 6x-His-NS2B/NS3protease in its native form was filtered (45 μm), batch-bound to 2 x 2 ml Ni^2+^-NTA (nickel-nitrilotriacetic acid) resin (pre-equilibrated with column buffer) and incubated overnight at 4°C. The resin was cleaned up from the unbound fraction by centrifugation and the resin with bound protein was collected and loaded into columns (Bio-Rad; 1 x 3 cm). The gradient technique columns were washed extensively with 3 x 15 ml of wash buffer and further eluted with 10 ml of elution buffer for each column while being monitored using Bio-Rad Bradford protein assay. The purified protein was then analysed with 12% SDS-PAGE, pooled and stored at -80°C for further use in the dengue protease activity and inhibition studies. The dengue protease activity assay was developed as previously described [51] with a slight modification [46,47,50]. Briefly, the assay system comprised of 200 mM Tris-HCl (pH 8.5) buffer, DENV-2 NS2B/NS3pro and Boc-GRR-MCA as the substrate. Protease optimum assay was executed to ascertain maximum protease activity at constant concentration of the substrate (25 μM). The protease concentrations were varied within 0–10 μM. The 7-Amino-4-methylcoumarin (AMC) [51] produced was measured as fluorescence intensity at λ_excitation_ of 340 nm and λ_emission_ of 440 nm by using ELISA modulus microplate reader.

The compounds were also checked for its requirement to pass the pan assay interference compounds (PAINS) (http://cbligand.org/PAINS/) before tested for their inhibition activities against DENV-2 NS2B/NS3pro at a range of concentrations (0 to 300 μg/mL). The result shows that all the tested compounds, except for **D1855** passed the PAINS filter. The concentration of the protease being used was 3 μM initially incubated with the compound for 10 minutes at 37°C with 200 rpm of rotation. Subsequently, 25 μM of the substrate was added and the incubation was further prolonged for 60 minutes. The fluorescence intensity at 340/ 440 nm was measured as the AMC byproduct was released upon the peptide substrate cleavage by the protease. The experiments were triplicated and Panduratin A was used as the positive control.

### Synthesis of thioguanine derivatives

All reactions were carried out using standard techniques for the exclusion of moisture [52,53] except those in aqueous media. The progress of reaction was monitored using TLC on 0.25 mm silica F_254_ and detected under UV light, or iodine vapor. ^1^H-NMR and ^13^C-NMR spectra were determined using Bruker Avance 500 spectrometer with TMS as an internal standard and the mass spectra were determined using XEVO-G2TOF #YCA153. Melting points were obtained using a STUART SMP electro-thermal apparatus and were uncorrected. Anhydrous reactions were carried out in over-dried glassware under a nitrogen atmosphere. The detail method of synthesis, the compounds’ structures as well as the numbering due to NMR assignation can be seen in S1 Text.

### Molecular docking simulation

To obtain the bound pose of the thioguanine analogues for MD simulation, molecular docking was carried out. 3D structures of the two most active compounds (**18** and **21)** were constructed and minimised using Hyperchem 8.0 [54] with 1000 steps of steepest descent followed by 1000 steps of conjugate gradient. The minimised structures were than subjected to molecular docking using AutoDock4.2 [55]. The same DENV-2 NS2B/NS3pro model [43] used in the virtual screening was used here. The protein and its inhibitors (compounds **18** and **21**) were subsequently prepared using AutoDockTools 1.5.6. Polar hydrogen and Kollman charges were added into the DENV NS2B/NS3pro. In preparing both the ligands, the non-polar hydrogen atoms were merged and Gasteiger charges were assigned. The grid box size and the grid spacing were set around the catalytic triad to 60×60×60 dimension and 0.375 Å respectively, with the centre set at x = 21.517, y = 43.428 and z = -1.743. AutoDock4.2 was used to run docking with the Lamarckian Genetics Algorithm (GA) search program applied to generate 100 runs. The conformations with the ones of lowest free energy of binding and of the most populated cluster were selected. The docked conformation for each compound (**18** and **21**) was used as the starting structure for the subsequent dynamics studies. The interaction analysis was conducted using ligplus+ [56] and VMD 1.9. Panduratin A was used as the positive control and prepared for the simulation, in a similar way as with compounds **18** and **21**.

### Molecular dynamics simulation

To gain insight into the binding interaction of compounds **18, 21** and panduratin A with NS2B/NS3pro, molecular dynamics simulations were carried out using Amber 14 [57]. The following is the description of the setups of NS2B/NS3pro-**18** (compound **18**), NS2B/NS3pro-**21** (compound **21**) and NS2B/NS3pro-panduratin. All systems were prepared using LEaP program. Amber generalised force filed (GAFF) were assigned to the three compounds whilst amber ff14SB to NS2B/NS3pro. Each system was neutralised with sodium ions and solvated using TIP3P water in a 53.57, 46.51, 44.05 Å truncated octahedral water box. To eliminate the steric clashes, each system was subjected to a total of 500 stepwise minimisation using steepest descent followed by conjugated gradient. The solvent of the system was first heated to 100 K with an NVT ensemble, followed by heating of the whole system to 300 K with an NPT ensemble. Throughout the MD simulation, a 0.2 fs time step, SHAKE algorithm, periodic boundary, and 10 Å cutoffs were applied. The analysis for RMSD and gyration was taken from the beginning. The equilibration phase was 1 ns, and the production phase of the simulation was 69 ns, with a total simulation time of 70 ns for each system. Analysis was conducted using CPPTRAJ modules in AmberTools 14 and visualised through VMD.

### MM/PBSA calculation

A total of 100 frames were extracted from the last nanosecond of the 70 ns simulation trajectory files. MM/PBSA calculations were performed on each frame using MMPBSA.py module in AMBER 14. Energy compositions of compounds **18**, **21** as well as panduratin A with NS2B/NS3pro was dissected accordingly.

## Results and discussion

### Virtual screening and the confirmation of activity of the *in silico* hits

**Fig 1:**
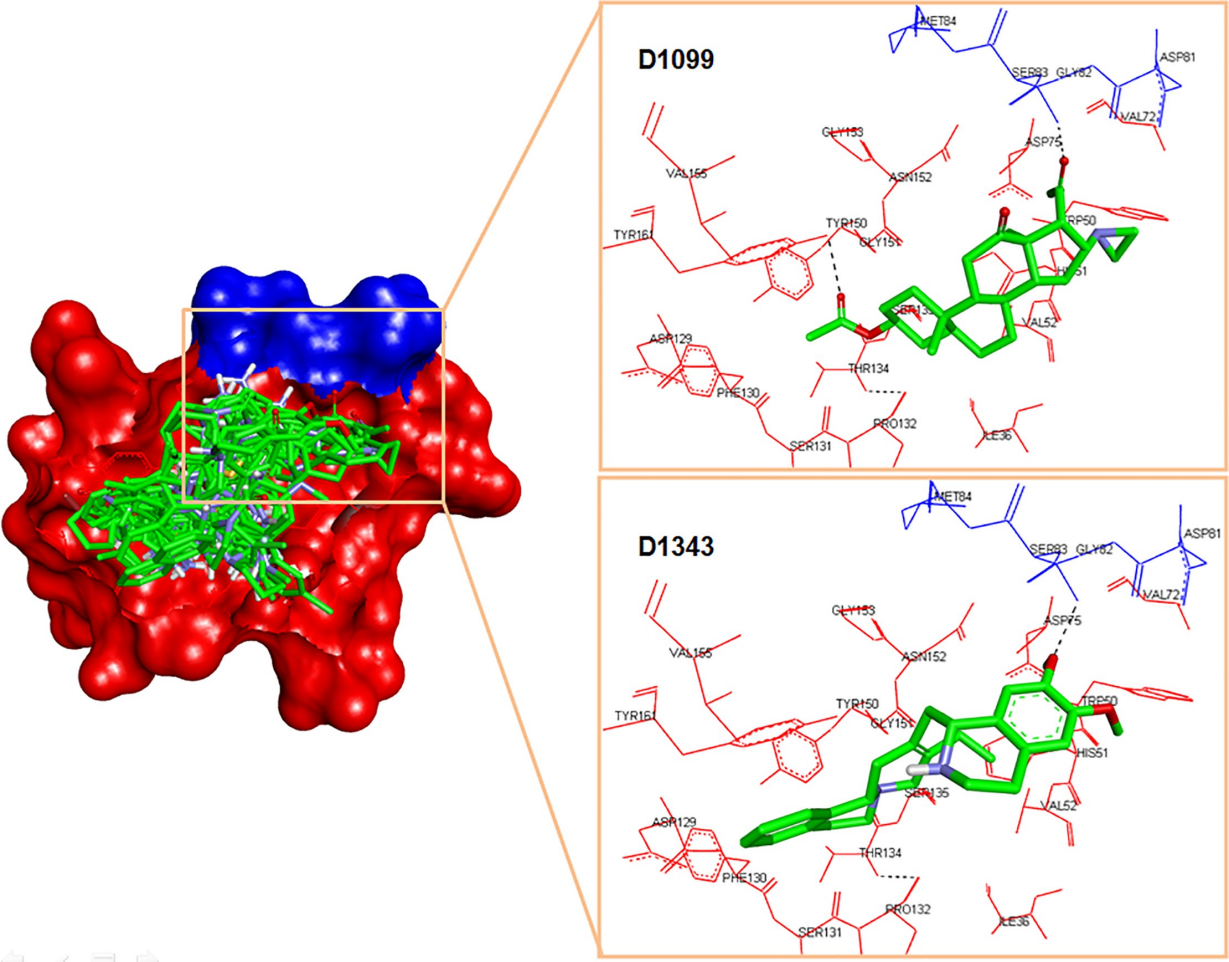
Docked conformation of top 24 NCI compounds in the active pocket of DENV-2 NS2B/NS3pro. The NS2B and NS3 domains are presented as surface form (blue area = NS2B, red area = NS3pro). Insets are the two ligands that bound to the NS2B, instead of NS3pro.

In this study, 1990 compounds from NCI Diversity Set II were docked into the active site pocket of DENV-2 NS2B/NS3pro. The active site is made up of important amino acid residues in S1-S4 subpockets such as Asp129_NS3, Ser135_NS3, Tyr150_NS3 and Tyr161_NS3 (S1 pocket); Asp81_NS2B, Gly82_NS2B, Ser83_NS2B, Asp75_NS3 and Asn152_NS3 (S2); Ser85_NS2B, Ile86_NS2B and Lys87_NS2B (S3); Val154_NS3 and Ile155_NS3 (S4). The 24 top hit compounds were investigated for their interaction with the active site’s residues and ranked in term of the free energy of binding (ΔG_bind_ = -10 kcal/mol to -5 kcal/mol, see S1 Table. We identified that there are more than 50 ligands docked into the active site with that ΔG_bind_ range, but only 24 of them shows interesting interactions with the essential amino acid residues. The selection of ΔG_bind_ range is adopted from the study reported by Shityakov (2014) that Gibbs free energy of binding < 6.0 kcal/mol is clustered as active when this prediction is highly correlated with the experimental results with R^2^ = 0.880; F = 692.4 standard error of estimate = 0.775 and p-value = 0.0001 [58]. This virtual screening study has a limitation in which no decoy database is being used to validate the docking protocol. The non-binding molecule could be selected over the true ligand called as false positive. Decoy database is the false positive hits which may improve the validity of the test by generating the true positive rate as well as enrichment factor. Unfortunately, the decoy for this DENV-2 NS2B/NS3pro has not been available in a database of useful decoy (DUD dude.docking.org), therefore, the validation of this virtual screening only relies on internal control docking and by *in vitro* assay [59]. Fig 1 shows the overlay of all 24 compounds docked into the active site of the protease. Most of the ligands bound to NS3pro, but there are two ligands (**D1099** and **D1343**) also interacting with NS2B, particularly at Ser83.

### Dengue protease inhibition assay

**Fig 2:**
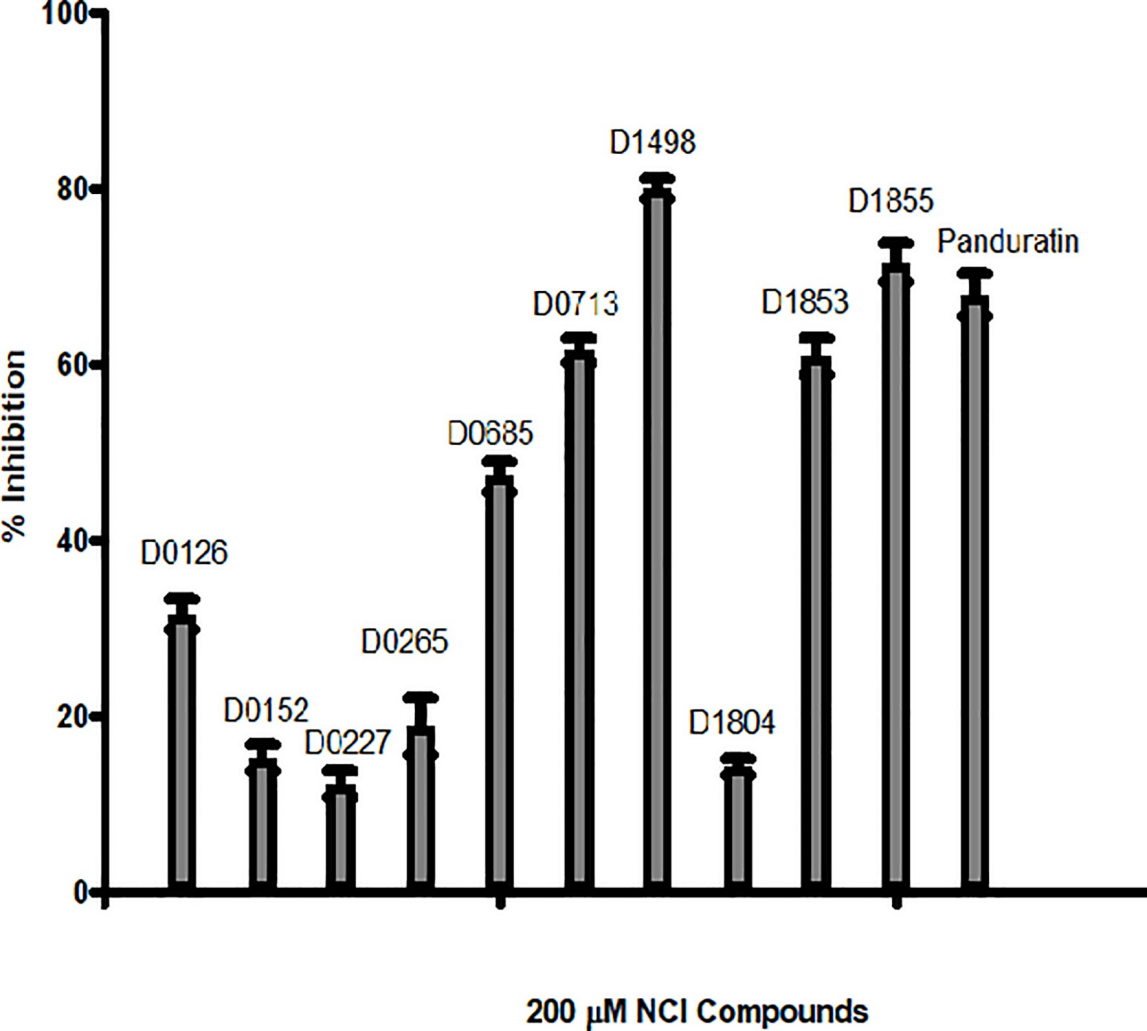
*In vitro* DENV-2 NS2B/NS3pro inhibition assay of Panduratin A and selected NCI compounds; NCI code D0265, D0685, D0227, D0152, D0126, D1804, D1855, D1498, D0713 and D1853 with D in the code stands for Diversity. The assays contained 0 μM NCI compounds were taken as 0% protease inhibition.

From the 24 hits identified in the virtual screening, only 20 were available for the inhibition assay. Of these 20 compounds, only 10 compounds exhibited significant inhibition activity towards DENV-2 NS2B/NS3pro activity (Fig 2). The other 10 NCI compounds exhibited negligible inhibition (<5% inhibition at 200 μM) towards the protease activity.

**Fig 3:**
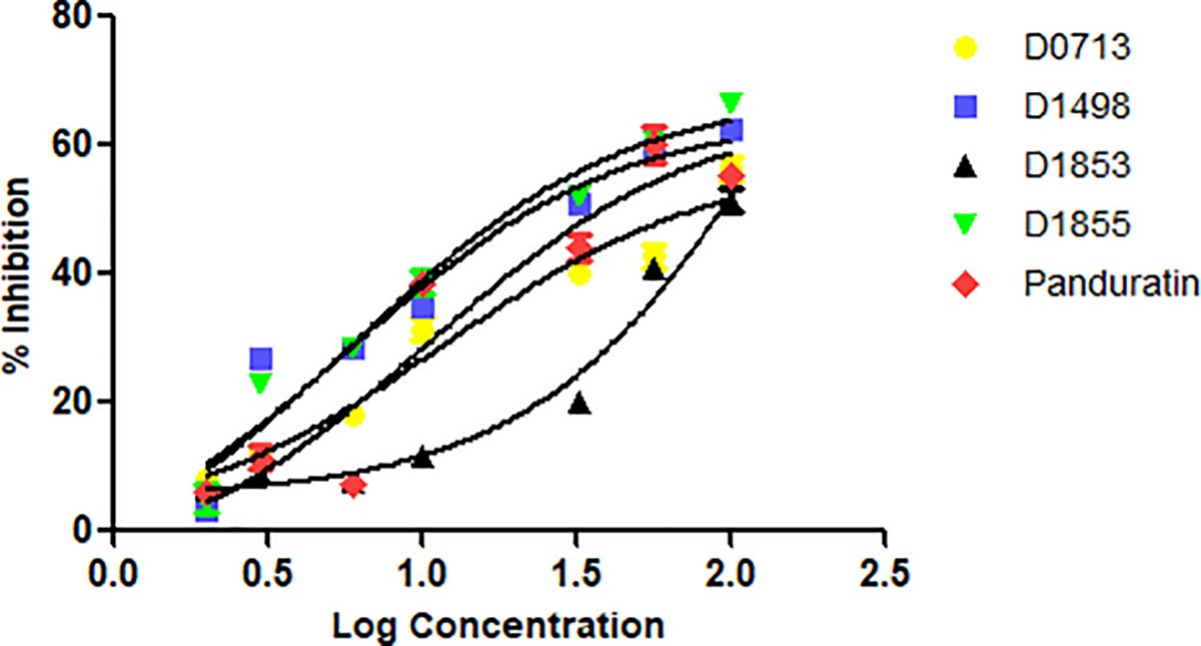
Plot of % DENV-2 NS2B/NS3pro inhibition vs log concentration of the four NCI compounds. Panduratin A was used as a control in this experiment.

Four NCI compounds with the percentage of inhibition greater than 50% were selected for further assay to determine their IC_50_ (Fig 3). **D1855** showed the strongest inhibition towards the protease activity with IC_50_ = 29 μM followed by **D1498** (48 μM), **D0713** (62 μM) and **D1853** (77 μM) (Table 1). Panduratin A was used as a control in this experiment. Previously, panduratin A was isolated from finger root (*Boesenbergia rotunda* (L.)) demonstrating competitive inhibition toward DENV-2 NS2B/NS3pro with K_i_ = 25 μM [31].

**Table 1:** Top *in vitro* hits from NCI diversity set compounds towards both S1 and S2 pockets of DENV-2 NS2B/NS3pro.

Ligands	ΔG_bind_ (Kcal/ mol)	Experimental IC_50_ (μM)
**D1498**	-7.40	48
**D1853**	-9.90	77
**D1855**	-8.90	29
**D0713**	-7.10	62
Panduratin A	-6.30	56

**Fig 4:**
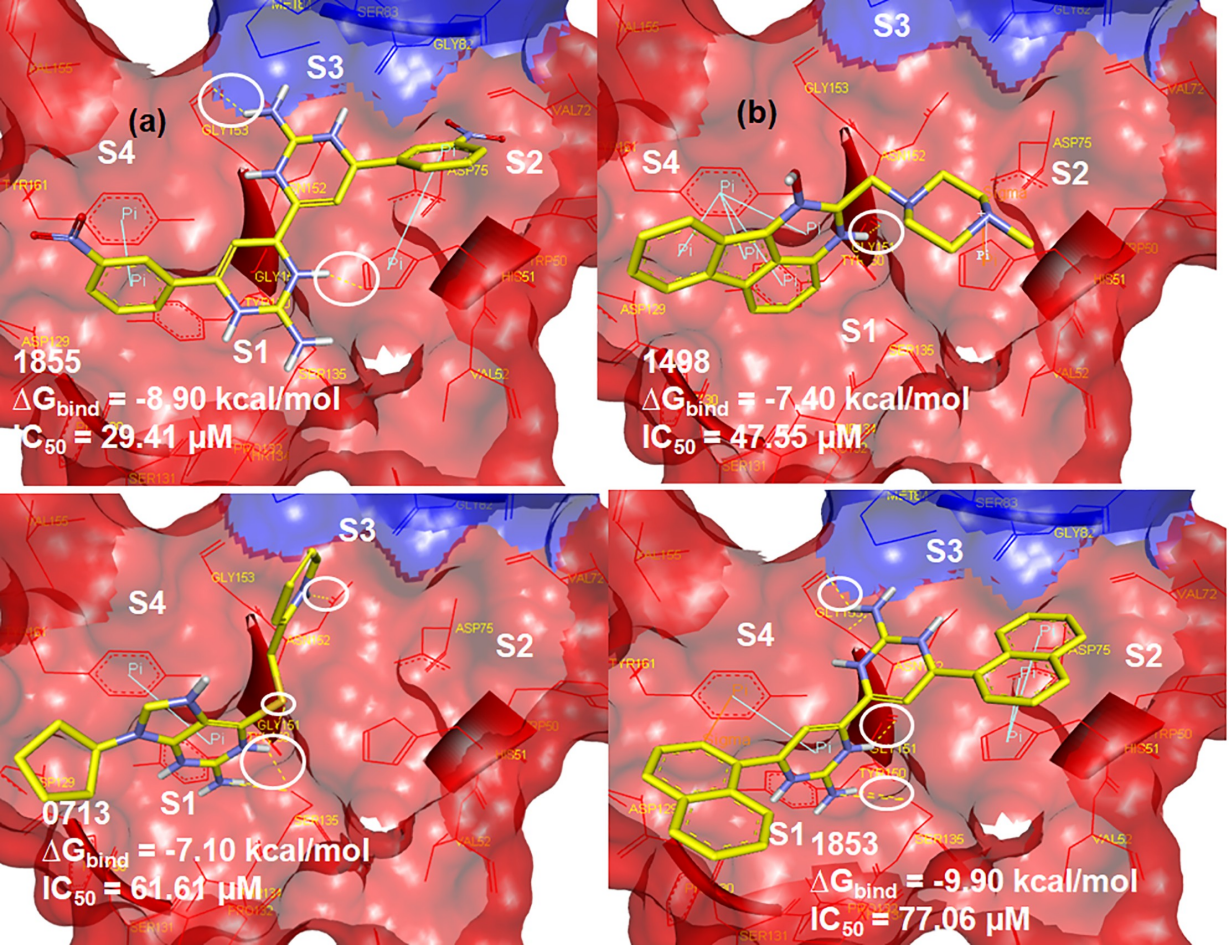
Docked poses of (a) **D1855**, (b) **D1498**, (c) **D0713** and (d) **D1853**. The NS2B and NS3pro domains are presented as surface form (blue area = NS2B, red area = NS3pro). The H-bond interactions are assigned as yellow dots inside the white circles.

**D1855** demonstrated the best inhibition towards the protease activity at concentration less than 50 μM as the relative protease activity dramatically decreased from 100 to 30% (IC_50_ = 29 μM). This inhibition is probably provided by the hydrogen bonding with Gly153 and His51 and the phenyl ring of the compound formed π-π- interactions with Tyr161 and His51 (Fig 4(A)). **D1498** (IC_50_ = 48 μM) is the second most potent of these four NCI compounds where the activity might be contributed by H-bond interaction with Gly151 of NS3, π—π interaction between its anthracene ring with Tyr161 and π-cation interaction with His51 (Fig 4(B)). **D0713** also demonstrated significant inhibition towards the protease with IC_50_ = 62 μM. The docked pose shows the formation of H-bonds between its amino groups with Asn152, Gly151, and Ser135 and π—π interaction between its guanine ring and Tyr161 (Fig 4(C)). Although the IC_50_ of **D1853** is approximately 77 μM, this compound showed nearly 70% inhibition of the protease activity at 200 μM. As with the above NCI compounds, this ligand also formed π- π as well as π-cation interactions with Tyr161 and His51, respectively, in addition to the H-bond interaction between its amino groups with Gly151, Ser135 and Gly153 (Fig 4(D)).

In our quest to identify suitable scaffold for designing potential novel and potent dengue protease inhibitors, we took into consideration the structure of **D0713**. Although this is not the most potent compound identified in the virtual screening, the structure contains thioguanine (**TG** or 6-thioguanine) scaffold. **TG,** a known drug used with other compounds in treating leukemia [40] has also been investigated in other pharmacological activities such as in autoimmune diseases [41] and transplant graft rejection [42]. In addition, its simple chemical structure also benefits feasible synthetic steps, thus, **TG** is selected as a template to develop a series of analogues. Therefore, in order to test our hypothesis, **TG** was subjected to the protease inhibition assay and it was found that the molecule showed 56% inhibition at 200 μg/mL (1 mM) indicating that even without any modification to the structure, the scaffold itself is able to inhibit the protease activity. Thus, **TG** was chosen as the lead structure in the design and synthesis of potential anti-dengue compounds.

### Design, synthesis and protease inhibition activity of thioguanine derivatives

**Fig 5:**
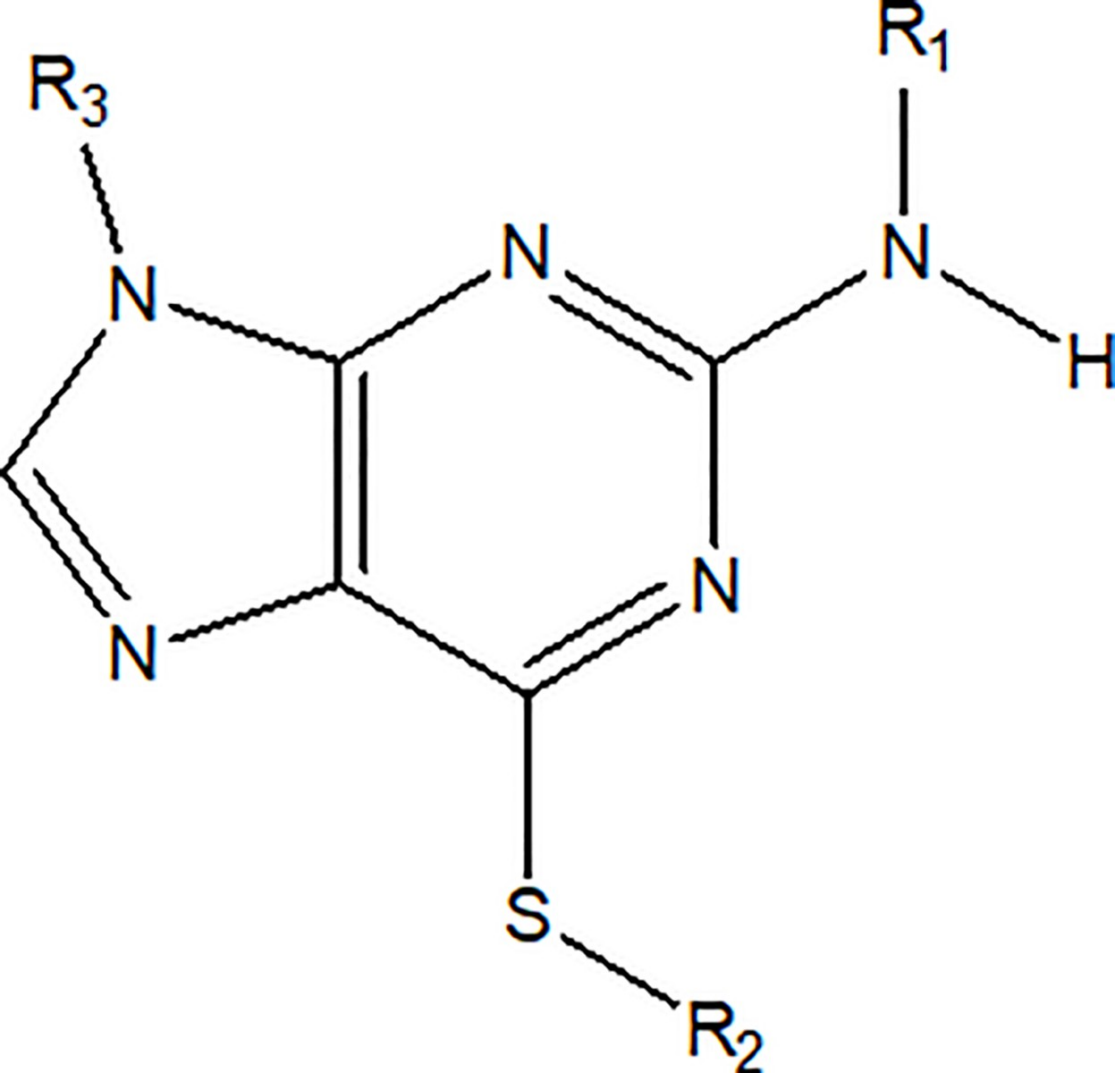
The structure of thioguanine scaffold.

The various thioguanine derivatives designed are shown in Table 2 with the scaffold being illustrated in Fig 5. The design was initiated by connecting the aromatic hydrophilic group as hydrogen bond acceptor (HBA) (**1–3)** with the amino group; and aromatic hydrophobic group with sulfonyl in **4–6** at R_1_. In **7–13,** acyl groups such as acetyl, butanoyl, isobutanoyl, pentanoyl, isopentanoyl and hexanoyl groups were placed at the same position, while benzoyl group was instead placed in **14**. Replacing aliphatic hydrophobic group at R_3_ with cyclopentyl group, as present in **D0713**, yielded **15**.

**Table 2:** The list of TG derivatives with their experimental IC_50_ against DENV -2 NS2B/NS3pro.

Ligands	R_1_	R_2_	R_3_	IC_50_ (μM)
**1**	(4-nitrophenyl)methanimine	H	H	1995
**2**	4-(iminomethyl)benzoic acid	H	H	1367
**3**	4-(iminomethyl)-2-methoxy-6-nitrophenol	H	H	28
**4**	3-methoxybenzenesulfinic acid	H	H	68
**5**	4-methoxybenzenesulfinic acid	H	H	55
**6**	3-methylbenzenesulfinic acid	3-methylbenzenesulfinic acid	H	63
**7**	acetyl	H	H	151
**8**	butanoyl	H	H	57
**9**	isobutanoyl	H	H	3893
**10**	pentanoyl	H	H	1037
**11**	3-methylbutanoyl	H	H	80
**12**	hexanoyl	H	H	168
**13**	palmitoyl	H	H	132
**14**	benzoyl	H	H	370
**15**	acetyl	H	cyclopentyl	556
**D0713**	H	2-methylpyridine	cyclopentyl	62
**TG**	H	H	H	753
**Panduratin A**	-	-	-	56

Compounds **1**–**3** are imine derivatives of **TG**. Imine itself has an electron withdrawing nature, albeit weakly. Attaching another EWG such as NO_2_ (**1**) and COOH (**2**) to the benzimine (in the *para* position) as R_1_ rendered the compounds to be inactive (IC_50_> 1000 μM). Interestingly, attaching an electron donating group (EDG) to benzimine at R_1_ (**3**) showed high protease inhibition (IC_50_ = 28 μM). These raised the idea to maintain the EWG group as a linker between the two aromatic rings of **TG** and the phenyl group which was modified to have EDG character.

Compounds **4** and **5** were designed to have sulfonyl (EWG) with CH_3_ and/ or OCH_3_ (EDG) on benzenesulfonyl as R_1_. These compounds demonstrated lower activities than **3** with IC_50_ of 68 and 55 μM, respectively. In **6,** the same group attached to R_1_ was also placed at R_2_ during the synthesis but changes in activity were insignificant. Placing at R_1_ with acetyl or extended alkyl chain (**7–13**), in general resulted in marked reduction in protease inhibition, with the exception noted for **11** which showed IC_50_ = 80 μM.

Placing a simple aromatic hydrophobic group (benzoyl) as seen in **14** did not increase the activity. Exploring placement at R_3_ with an aliphatic hydrophobic group (**15**) also did not produce a compound with any improvement in the protease inhibition activity. At this point, it was decided to synthesize six more compounds with expected higher activities.

**Fig 6:**
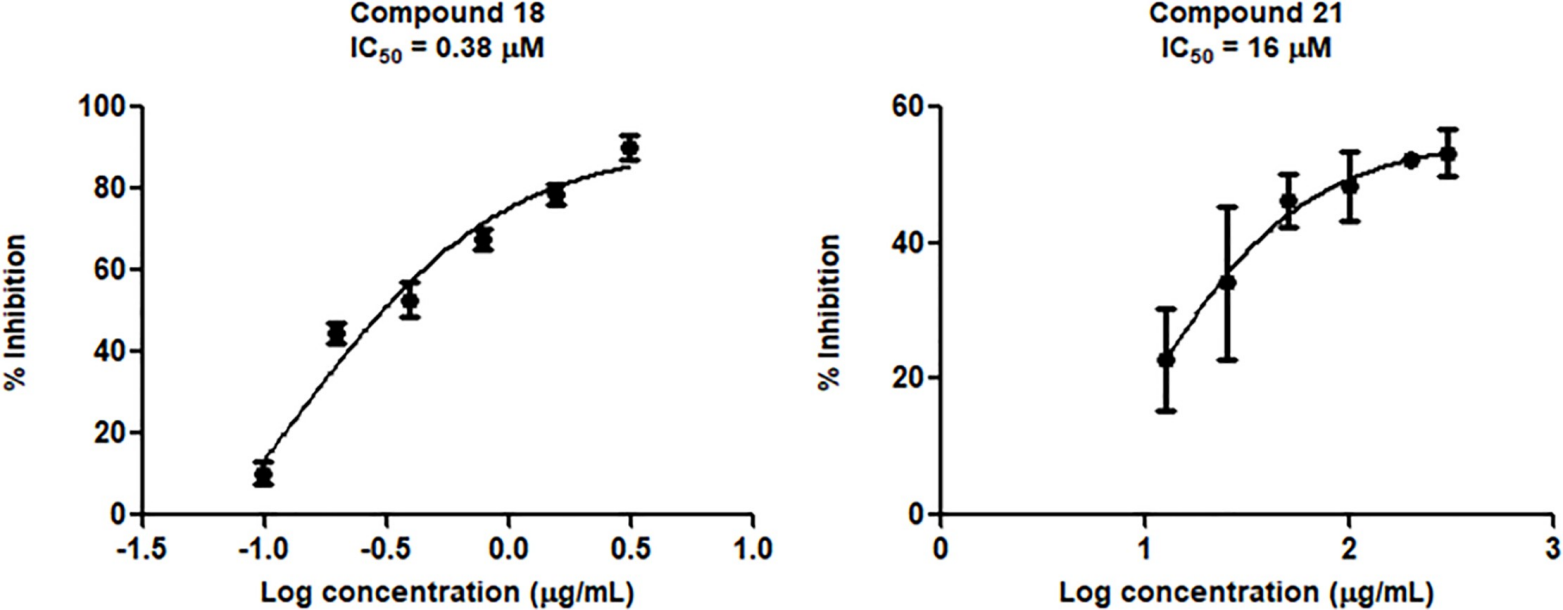
The log dose dependent curve of Compound 18 and 21 against DENV-2 NS2B/NS3pro.

Inspired by **7**, **8** and **11** where the compounds have amide group with promising inhibitory activity, six compounds (**16–21,** see Table 3) having amide group with diverse alkyl chains were designed. As the natural substrate of the protease is peptide, it made sense to incorporate amide groups into the inhibitor structure in order to mimic the peptide character. High activity of **18, 19 and 21** towards DENV-2 NS2B/NS3pro (IC_50_ of 0.38, 54 and 16 μM, respectively) could be due to this mimic of peptide character. Compound **18** with pentanamide chains at both R_1_ and R_2_ possessed the highest activity against the protease. However, attaching benzyl group at R_2_ (**19**) dramatically decreased the activity from 0.38 to 54 μM. Interestingly, attaching benzyl group at R_3_ significantly increased the activity to 16 μM. Compounds **16** and **17** have amide group extended by shorter alkyl chains showed moderate activity with IC_50_ = 97 and 80 μM, respectively. To confirm the essentiality of amide group, in compound **20**, the amide group at R_1_was omitted and at R_2,_ it was replaced with methylnaphthalene whilst at R_3_ with isopropyl chain. As predicted, the activity of **20** significantly decreased with IC_50_ = 258 μM. The activity-dose curve of compounds **18** and **21** are presented in Fig 6.

**Table 3:** The structures of six newly designed compounds and their activity against DENV-2 NS2B/NS3pro.

Ligands	R_1_	R_2_	R_3_	IC_50_ (μM)
**16**	propanoyl	propanoyl	H	97
**17**	isopropanoyl	isopropanoyl	H	80
**18**	pentanoyl	pentanoyl	H	0.38
**19**	pentanoyl	benzyl	H	54
**20**	H	2-methylnaphtyl	isopropyl	258
**21**	pentanoyl	benzyl	benzyl	16

### Molecular docking simulation

**Fig 7:**
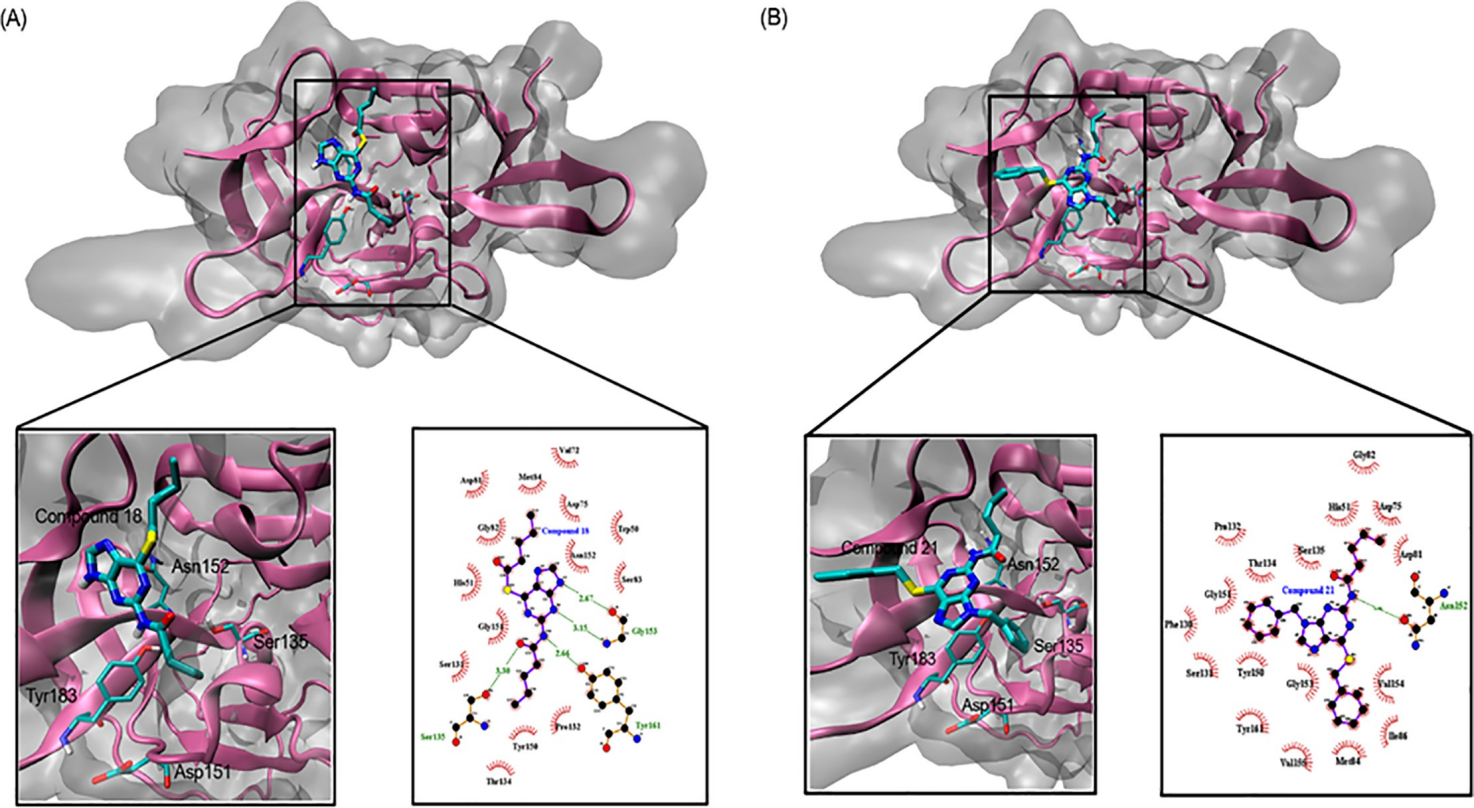
Binding orientation and interaction mode of compound with compound **18** (A) and compound **21** (B) in molecular docking simulation.

From the docking results, several interactions were identified between NS2B/NS3pro with **18** and **21**. For **18**, interacting residues such as Ser157(135), Tyr183(161) and Gly175(153) indicated potential hydrogen bonding interactions. The numbering in the bracket indicates the numbering in Wichapong model. Binding stability is contributed by all the hydrophobic interactions surrounding the entire binding site with the free energy of binding– 7.49 kcal/mol (Fig 7). In addition, **21** demonstrated binding affinity (ΔG = -8.10 kcal/mol) with the hydrogen bonding also observed with Asn174.

### Stability and conformational changes in MD complexes

**Fig 8:**
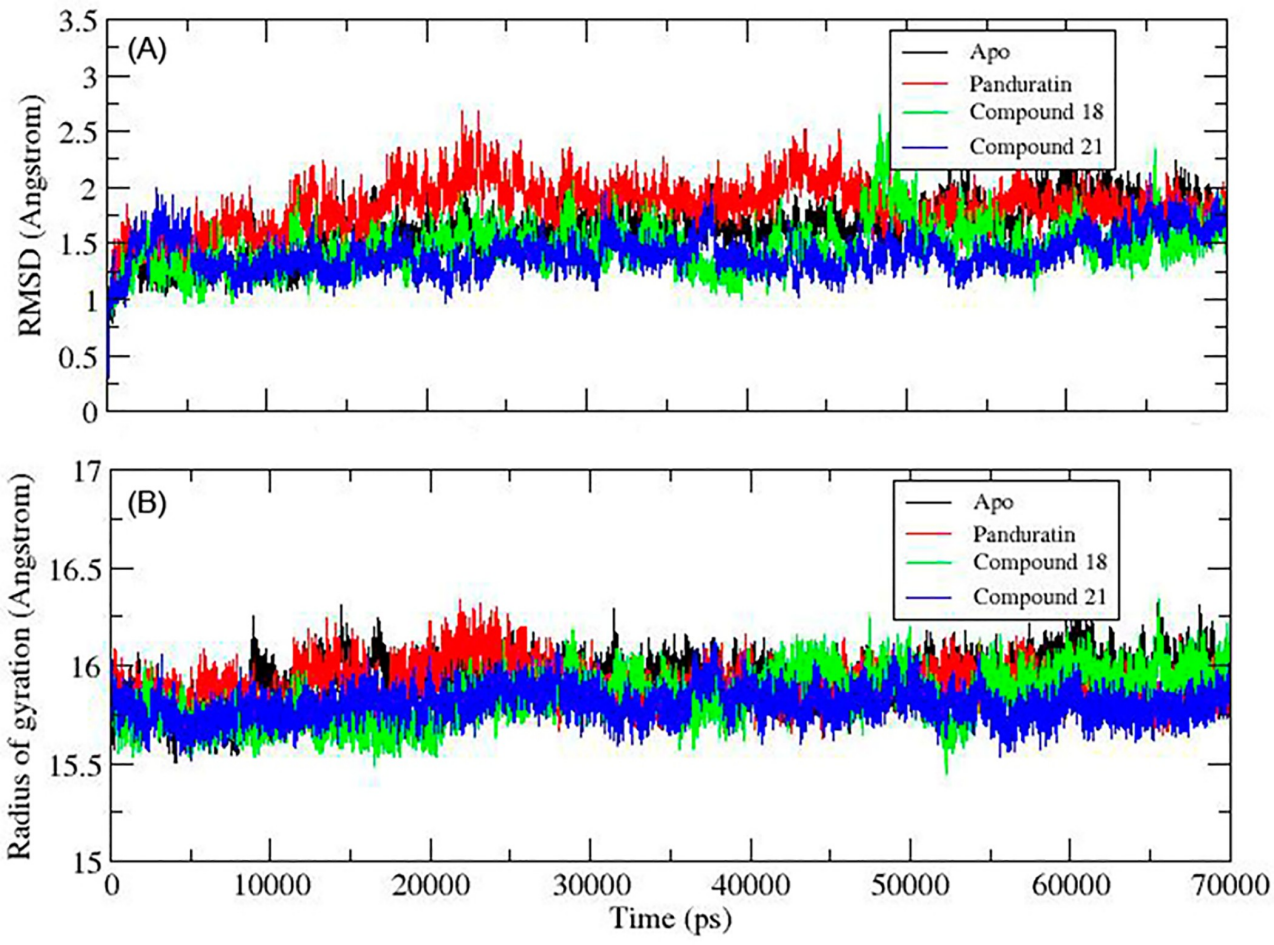
(A) Time evolution of RMSD of NS2B/NS3pro backbone CA unbounded (Apo) (black), bounded with panduratin A (red), **18** (green) and **21** (blue). (B) Time evolution of radius of gyration of NS2B/NS3pro backbone CA unbounded (Apo) (black), bounded with panduratin A (red), **18** (green) and **21** (blue).

To further substantiate the stability and conformational changes, MD simulation was carried out to understand the dynamic features of the two best compounds with respect to time at nanosecond scale. Overall the backbone RMSD values for Apo, NS2B/NS3pro-**18**, NS2B/NS3pro-**21** are <2 Å throughout the 70 ns simulation time reflecting the stability of the systems. NS2B/NS3pro-Panduratin adopted a higher RMSD with the range of 2.0 to 2.5 Å at 20 to 50 ns simulation time, however, it stabilised at <2 Å from 50 to 70 ns of simulation time (Fig 8).

The gyration value indicates the compactness of the protein which in turn reflects directly on the folding and unfolding of the protein where unfolding of the protein would affect the kinetic of the protein activity. The gyrations of all the four systems (apoenzyme, **18**, **21** and Panduratin A) are fluctuating in less than 1 Å at 15.5 to 16.5 Å. No unfolding is observed with a low fluctuation on the gyration.

### Hydrogen bonding analysis

Protein-ligand interaction in general is stabilised by various types of weak interactions with hydrogen bonding interaction is potentially one of the most important interactions. Hydrogen bond formation is observed with several important interacting residues such as His73(51), Asp151(129), Ser157(135), Asn174(152) and Tyr183(161) in both systems (Table 4) (The number in parenthesis is according to Wichapong model). Asn174 in NS2B/NS3pro played an important role in forming hydrogen bonds which occupied more than 60% of the simulation time with **18**. Asp97(75) was also found to form hydrogen bonds with both nitrogen groups of **18** with the total occupancy of 8.22% simulation time. It is interesting to note that Tyr183(161) and Ser157(135) which are important in the protease activity also demonstrated weak hydrogen bonds formation with the occupancy of 2.85% and 2.73%, respectively. It is thus postulated that these hydrogen network together with the hydrophobic clusters contributed to the binding affinity of **18**.

**Table 4:** Hydrogen bonds between compound 18 and compound 21 with NS2B/NS3pro that found with at least 0.1% occupancy throughout 70ns simulation time.

Complex	Hydrogen bond formation	Distance (Å)	Occupancy (%)
NS2B/NS3-18	Gly173@O-Comp18@H5/Comp18@N4	2.85	27.19
	Asn174@OD1-Comp18@H25/Comp18@N2	2.85	21.16
	Comp18@N1-Asn174@HD21/Asn174@ND2	2.91	11.21
	Comp18@N1-Tyr183@HH/Tyr183@OH	2.83	8.33
	Gly175@O-Comp18@H25/Comp18@N2	2.85	6.41
	Asp97@OD1-Comp18@H25/Comp18@N2	2.82	4.46
	Tyr183@OH-Comp18@H5/Comp18@N4	2.90	2.74
	Comp18@O2-Ser157@HG/Ser157@OG	2.77	2.19
	Asp97@OD2-Comp18@H5/Comp18@N4	2.83	1.93
	Asp97@OD2-Comp18@H25/Comp18@N2	2.84	1.83
	Gly35@O-Comp18@H5/Comp18@N4	2.86	1.15
	Ser157@OG-Comp18@H5/Comp18@N4	2.89	0.54
	Ser36@OG-Comp18@H5/Comp18@N4	2.88	0.51
	Comp18@O2-His73@HE2/His73@NE2	2.86	0.45
	Asp34@O-Comp18@H5/Comp18@N4	2.85	0.36
	Gly175@O-Comp18@H5/Comp18@N4	2.85	0.23
	Comp18@O2-Ser36@HG/Ser36@OG	2.78	0.23
	Met37@O-Comp18@H25/Comp18@N2	2.84	0.22
	Ser36@OG-Comp18@H25/Comp18@N2	2.90	0.15
	Comp18@N9-Ser36@HG/Ser36@OG	2.87	0.13
	Comp18@O2-Tyr183@HH/Tyr183@OH	2.81	0.12
	Gly35@O-Comp18@H25/Comp18@N2	2.88	0.12
	Tyr183@OH-Comp18@H25/Comp18@N2	2.92	0.11
	Asn174@OD1-Comp18@H5/Comp18@N4	2.94	0.10
	Comp18@O1-Arg76@HH22/Arg76@NH2	2.89	0.10
NS2B/NS3-21	His73@ND1-Comp21@HN/Comp21@N	2.89	27.40
	Ser157@OG- Comp21@H1/Comp21@N1	2.86	5.12
	Comp21@O-Ser157@HG/Ser157@OG	2.79	4.58
	His73@ND1-Comp21@H/Comp21@N4	2.82	0.96
	Asp97@OD2-Comp21@HN/Comp21@N	2.84	0.39
	Asp97@OD1-Comp21@HN/Comp21@N	2.82	0.25
	Gly175@O-Comp21@HN/ Comp21@N	2.85	0.15
	Asn174@OD1- Comp21@H/Comp21@N4	2.82	0.12
NS2B/NS3-panduratin	Phe152@O-Pandu@H10/Pandu@O3	2.68	9.59
	His73@O-Pandu@H9/Pandu@O1	2.71	5.74
	Phe152@O-Pandu@H9/Pandu@O1	2.71	1.50
	Pandu@O-Tyr183@HH/Tyr183@OH	2.79	1.29
	Asp151@OD2-Pandu@H9/Pandu@O1	2.65	1.13
	Asp151@OD1-Pandu@H9/Pandu@O1	2.67	0.57
	Pandu@O2-Tyr172@HH/Tyr172@OH	2.86	0.41
	Pandu@O2-Val177@H/Val177@N	2.89	0.40
	Pandu@o2-Ser157@HG/Ser157@OG	2.83	0.28
	Pandu@O3-Tyr183@HH/Tyr183@OH	2.86	0.22
	Pandu@O2-Tyr183@H/Tyr183@N	2.91	0.19
	Asp151@OD1-Pandu@H10/Pandu@O3	2.66	0.16
	Val74@O-Pandu@H9/Pandu@O1	2.80	0.13
	His73@ND1-Pandu@H9/Pandu@O1	2.85	0.13
	Pandu@O3-Phe152@H/Phe152@N	2.91	0.12
	Gly173@O-Pandu@H9/Pandu@O1	2.79	0.10
	Pandu@O-Tyr183@H/Tyr183@N	2.90	0.10

The number of hydrogen bond formation observed with **21** is lesser as compared to **18**. His73 formed a hydrogen bond with nitrogen group in **21** with 27.4% occupancy of the total 70ns simulation time. Two hydrogen bonds between Ser157 and the nitrogen and oxygen groups of **21** with 5.12% and 4.58% occupancy while very weak hydrogen bonds were observed with other interacting residues such as Asp97, Asn174 and Tyr183 with less than 1% occupancies.

In the case of panduratin A, only weak hydrogen bonds with less than 10% occupancies throughout 70ns were found with residues such as Phe152, His73, Asp151 and Tyr183. The formation of hydrogen bonding is significantly weaker as compared to **18** and **21**.

### Comparison of free energy of binding between panduratin A, 18 and 21

The interaction and decomposition energies of the interactions between panduratin A, **18** and **21** were calculated using MM/PBSA. A total of 100 snapshots were extracted every 10 ps interval from the last nanosecond. The absolute binding free energy (Table 5) for panduratin A, **18** and **21** are-11.27 ±2.99, -16.10 ± 2.70 and -18.24 ± 4.66 kcal/mol, respectively. Non-polar interaction between the binding site residues as well as the aliphatic chain (pentanoyl) of **18** and **21** provides major contribution to the binding energy. The binding of panduratin A against NS2B/NS3pro is less favourable as compared to **18** and **21**. Similarly, major contribution of panduratin A binding is mainly from the non-polar interaction. This can be inferred from the fact that only weak hydrogen bonds were found between panduratin A and NS2B/NS3pro with low occupancies. This reflects that the binding of panduratin A is stabilised mainly by van der Waals and hydrophobic interactions.

**Table 5:** Binding free energy predicted using MM/PBSA calculation for 18, 21 and panduratin A.

Energy Component	Binding Free Energy (kcal/mol)		
	Compound 18	Compound 21	Panduratin A
Polar component electrostatics	-30.05 ± 8.77	-264.23 ± 17.87	-1.90 ±6.37
Electrostatics solvation	42.54 ± 8.73	292.37 ± 17.63	14.21 ± 4.22
Non-polar component van der Waal	-26.22 ± 2.6	-44.36 ± 2.83	-21.18 ± 2.54
Non-polar solvation	-2.66 ± 0.15	-4.73 ± 0.10	-2.40 ±0.17
TOTAL Binding Free energy	-16.37 ± 3.22	-20.95 ± 4.12	-11.27 ± 2.99

The binding free energy estimated by MMPBSA calculation indicated that **21** has better binding as compared to **18** but the IC_50_ for **18** is better. **18** and **21** have identical pentanoyl R_1_ group but **21** has two aromatic benzyl groups at R_2_ and R_3_ instead of a carbonyl chain and hydrogen for **18** at R_2_ and R_3_, respectively. Higher van der Waals interaction was observed from MM/PBSA calculation for **21** due to the presence of these two benzyl groups at R_2_ and R_3_. However, these interactions might be overestimated using MM/PBSA approach [60].

## Further discussion

Thioguanine was identified as a potential scaffold for DENV-2 NS2B/NS3pro inhibitor based on virtual screening, *in vitro* assay and molecular modelling. The thioguanine scaffold is composed of pyramidine and imidazole rings attached to an amine and a thiol group which may contribute to the compound’s activity. As far as we are aware, no report of pyrimidine inhibition to DENV-2 NS2B/NS3pro, however, 2-(benzylthio)-6-oxo-4-phenyl-1,6-dihydropyrimidine has been reported to demonstrate an activity against SARS-CoV-3C like protease [61]. In addition, pyrimidine has also been shown to actively stop the growth of DENV-2 but it relies on the capability of pyrimidine to inhibit dihydroorotate dehydrogenase (DHODH), an enzyme required for viral pyrimidine biosynthesis [62]. Imidazoles have also been investigated against dengue virus [63] however the exact molecular mechanism or which protein they target is still unknown. The 6-thioguanine scaffold has been reported to non-competitively inhibit ubiquitin specific protease in various cancers [64]. With this background, we postulated that thioguanine derivatives might potentially be good inhibitors against DENV-2 NS2B/NS3pro.

Two most active compounds (**18** and **21**) against DENV2-NS2B/NS3pro have an amide functional group (instead of an amino group). Amide is a part of peptide which is recognised as the key point for the protease substrate. In the docking study, the amide group is marked as a HBD with N-amide interacts with O-phenol of Tyr161 (in **18**) and Asn152 (in **21**) via H-bond. Previously, amide, such as α-ketoamide [65], arylcyanoacrylamide [66], and aminobenzamide [67] has been incorporated in the design of DENV NS2B/NS3pro inhibitors. The alkyl chain of **18** and phenyl-benzyl group of **21** demonstrate hydrophobic features and agreeable to docking results which show that these groups bound at the hydrophobic pocket surrounded by Val154, Ile86, Met84 and Val155. These also correlate with the decomposition energies as computed by MM/PBSA which highlight that the major contribution to the interaction with DENV-2 NS2B/NS3pro is from non-polar interaction.

**Fig 9:**
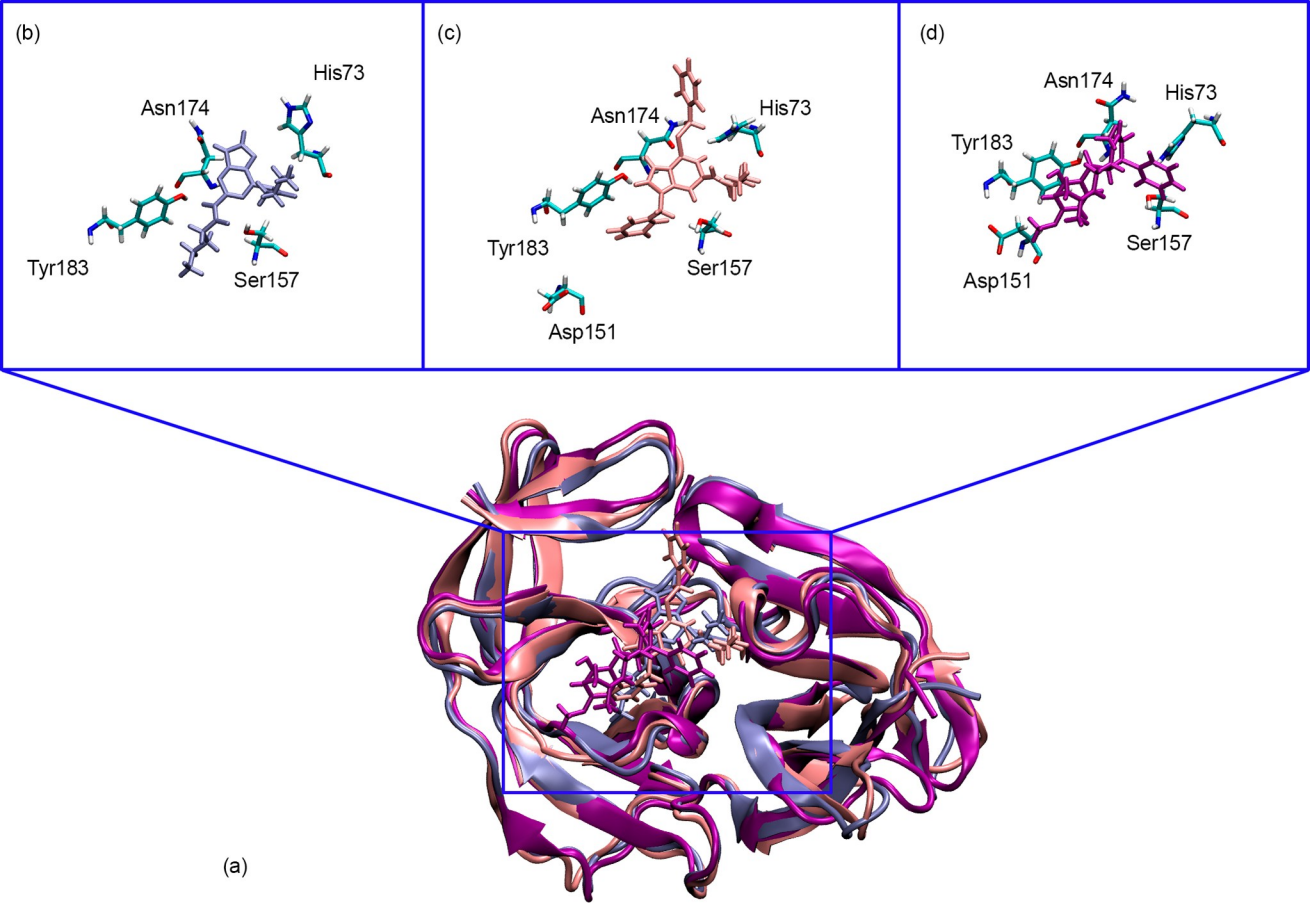
Binding orientation and interaction mode of compound with **18** (A), **21** (B) and panduratin A (C) in molecular dynamics simulation.

It is interesting to note that the binding orientations from the MD simulations for both compounds are different despite sharing similar thioguanine (Fig 9). The thioguanine moiety is facing toward His73 and Tyr183 for **18** and **21**, respectively. Compound **18** has an acyl group at R_2_ which provides additional electron lone pair enabling the formation of hydrogen bonds that can strengthen its binding towards NS2B/NS3pro. This reflected clearly in the hydrogen bond analysis where **18** formed more hydrogen bonds as compared to **21**. Regardless of the slightly lower absolute binding energy from the MM/PBSA prediction, the binding interaction analysis of **18** with NS2B/NS3pro agrees well with experimental results. The absolute free energies of binding for both two active compounds are also more negative than panduratin A supporting the *in vitro* results in which compound **18** and **21** better IC_50_ value (0.38 and 16 μM, respectively) than panduratin A (56 μM).

## Conclusions

We presented here the computational design of potential DENV-2 NS2B/NS3pro inhibitors. From the virtual screening of National Cancer Institute Database, four compounds including **D0713** were observed to have moderate inhibition activity on the protease. **D0713** has a thioguanine scaffold in its structure prompting us to consider the scaffold for designing the new thioguanine derivatives as potential DENV-2 NS2B/NS3pro inhibitors. Fifteen compounds were synthesised and the bioactivity against dengue protease showed variation in inhibition activity from inactive to moderately active (1000>IC_50_>18 μM). Based on this information, a further design of six new compounds was conducted by concentrating on the attachment of amide group. All the compounds showed inhibition activity against the dengue protease with compound **18** being the most potent (IC_50_ of 0.38 μM). This result agrees well with the MM/PBSA calculation which showed that the interactions are mainly contributed by polar and non-polar interactions. Hydrogen bonding analysis demonstrates the importance of amino acid residues Asn174 (occupancy 60%), Asp75 (8.22%), Tyr183 and Ser157 (2.85 and 2.73%). This is further supported by the experimental results which showed that **18** could be further developed as DENV-2 NS2B/NS3pro inhibitor. It is hoped that the results obtained from this study could be used in designing more active compounds as potential dengue protease inhibitors.

## Supporting information

S1 Text Synthesis of thioguanine derivatives. General procedure for synthesis of Schiff base-thioguanine compounds, acetamide-thioguanine compounds, *N*-alkylation of acetamide-thioguanine, and *S*/*N*-benzylation of acetamide-thioguanine.
(PDF)

S1 Fig Structure of 1–21. The numbering system is according to the NMR characterisation.
(PDF)

S2 Fig The molecular interaction between Panduratin A with DENV-2 NS2B/NS3pro. The NS2B-NS3pro is presented in a surface form. The ligands are presented in a stick form in which carbons are grey while oxygens are red. The ligand is docked at the active site of the protease with the hydrophilic moieties surrounded by catalytic site(His51, Asp75 and Ser135) whereas the nonpolar part of the ligand closes nearly to hydrophobic area of the protease wherein phenyl ring posseses H-bond as well as π-π interactions with Tyr161.
(PDF)

S1 Table The 24 NCI compounds selected from virtual screening. The NCI compounds are named in two codes and the free energy of binding upon DENV-2 NS2B/NS3pro binding site is expressed in kcal/mol.
(PDF)
