# Supplementary material for: Thioguanine-based DENV-2 NS2B/NS3 protease inhibitors: Virtual screening, synthesis, biological evaluation and molecular modelling
Source: PLoS One. 2019 Jan 24;14(1):e0210869. doi: 10.1371/journal.pone.0210869 (PMC6345492; doi:10.1371/journal.pone.0210869)
Supplement: S1 Text — General procedure for synthesis of Schiff base-thioguanine compounds, acetamide-thioguanine compounds, N-alkylation of acetamide-thioguanine, and S/N-benzylation of acetamide-thioguanine. (PDF) [file pone.0210869.s001.pdf]

Supporting Information

Thioguanine-based DENV-2 NS2B/NS3 protease inhibitors: Virtual screening, synthesis, biological evaluation and molecular modelling

Maywan Hariono[1,2¶], Sy Bing Choi[1,9&], Ros Fatihah Roslim[1&], Mohamed Sufian Nawi[1,3&], Mei Lan Tan[4], Ezatul Ezleen Kamarulzaman[1], Nornisah Mohamed[1], Rohana Yusof[5], Shatrah Othman[6], Noorsaadah Abd Rahman[6], Rozana Othman[7], Habibah A. Wahab[1,8*]

[1]School of Pharmaceutical Sciences, Universiti Sains Malaysia, Minden, Pulau Pinang, Malaysia

[2]Faculty of Pharmacy, Sanata Dharma University, Maguwoharjo, Sleman, Yogyakarta, Indonesia

[3]Department of Pharmaceutical Chemistry, Kulliyah of Pharmacy, International Islamic University Malaysia, Kuantan, Pahang, Malaysia

[4]Advanced Medical and Dental Institute, Universiti Sains Malaysia, Bertam, Pulau Pinang, Malaysia

[5]Department of Molecular Medicine, Faculty of Medicine, Universiti Malaya, Kuala Lumpur, Malaysia

[6]Department of Chemistry, Faculty of Science, Universiti Malaya, Kuala Lumpur, Malaysia

[7]Department of Pharmacy, Faculty of Medicine, Universiti Malaya, Kuala Lumpur, Malaysia

[8]Malaysian Institute of Pharmaceuticals and Nutraceuticals, Ministry of Science, Technology and Innovation, Halaman Bukit Gambir, Bayan Lepas, Pulau Pinang, Malaysia

[9]School of Data Sciences, Perdana University, Blok B and d1, MAEPS Building, MARDI Complex, Jalan MAEPS Perdana, 43400 Serdang, Selangor

[*]Corresponding Author
E-mail: habibahw@usm.my ; bibwahab@gmail.com

[&]These authors contributed equally to this work

S1 Text

General procedure for synthesis of Schiff base-thioguanine compounds

The synthesis of 1-3 followed the general procedure for Schiff Base reactionPannerselvam et al., 2005[1] . An amount of 6-thioguanine (0.6 mmol) was mixed with an equal mol number of the corresponding aromatic aldehyde and stirred up to 15 minutes. The liquid mixture of ethanol-NaOH 10% (1.5 mL (1:1)) was added to the first mixture and then the stirring was continued until the yellowish mixture formed. Next, the second volume of ethanol-NaOH 10% was added and the stirring was continued until the product was completely formed (monitored by TLC with $CHCl_3$ – Methanol (2:2) as a mobile phase). The mixture was neutralized using HCl 6 M and the solid product was filtered out using filter paper. The product was collected, washed with water, followed by washing the product using ethyl acetate and then recrystallised from a hot methanol to afford the pure product.

2-[(4-nitrobenzylidene)amino]-9H-purin-6-thiol (1)

Yellow powder, yield 52%, decomps. 280°C.; υ_{max}/ cm^{-1} (KBr) 3375 (NH; ^{1}H-NMR (DMSO-D_6) δ_H 8.18 (2H, *d*, J_{ortho}= 9 Hz, H14 and H18), 8.33 (2H, *d*, J_{ortho}= 9 Hz, H14 and H18), 8.79 (1H, *s*, H12), 12.55 (1H, *br*, *s*, H1); δ_C 120.8 (C15 and C17), 124.2 (C14 and C18), 131.1 (C4), 136.8 (C13), 140.8 (C2), 148.7 (C16), 150.5 (C9), 154.6 (C7), 166.2 (C12), 172.0 (C5);QTOF-MS m/z calcd for $C_{12}H_8N_6O_2S[M+3H]^+$303.07 found 303.50.

4-{[(6-sulfanyl-9H-purin-2-yl)imino]methyl}benzoic acid (2)

Yellow powder, yield 47%, decomps. 231-235°C; υ_{max}/ cm^{-1} (KBr) 2946 (NH), 1687 (C=O); ^{1}H-NMR ($CDCl_3$) δ_H 5.46 (1H, *s*, SH), 7.57 (2H, *d*, J_{ortho}= 8Hz, H14 and H18), 8.00 (1H, *d*, J_{ortho}= 8.5 Hz, H2), 8.12 (2H, *d*, J_{ortho}= 8.5 Hz, H15 and H17), 8.26 ((1H, *d*, J_{ortho}= 8.5 Hz, H12), 10.13 (1H, *s*, H1); δ_C 95.5 (C14 and C18), 196.0 (C15 and C17), 98.0 (C4), 126.9 (C19), 129.5 (C13), 130.1 (C2), 130.7 (C7),

134.4 (C9), 139.5 (C12), 168.3 (C19), 191.6 C5). QTOF-MS m/z calcd for $C_{13}H_9N_5O_2S[M+3H]^+$302.37 found 303.05.

2-methoxy-6-nitro-{[(6-sulfanyl-9H-purin-2-yl)]imino]methyl}phenol (3)

Light yellow powder; yield 81%; decomps. 280-289°C; υ_{max}/ cm^{-1} (KBr) 2987 (NH); ^{1}H-NMR (DMSO-D_6) δ_H 3.67 (3H, *s*, H20), 6.83 (1H, *d*, J_{meta}= 2.5 Hz, H14), 7.96 (1H, *d*, J_{meta}= 2Hz, H18), 8.32 (1H, *s*, H12); δ_C 55.5 (C20), 104.8 (C18), 116.2 (C13), 129.7 (C4), 135.4 (C22), 137.3 (C16), 156.2 (C2), 165.4 (C9), 188.3 (C15); QTOF-MS m/z calcd for $C_{13}H_9N_5O_2S[M+K]^+$385.41 found 385.24.

General procedure for synthesis of benzenesulfonamide-thioguanine compounds

The synthesis of **4-6** followed the general procedure for tosylationRamezanian et al., 1993[2]. The starting material, 6-thioguanine (0.6 mmol) was dissolved into 5 mL of NaOH 10% on an ice bath containing 3-methylbenzenesulfonyl chloride (0.7 mmol) and was carefully dropped and then the mixture was stirred at room temperature while the reaction's progress was monitored using TLC (*n*-hexane-ethyl acetate (2:2)). After the reaction completed, HCl 6M was added until the pH of the mixture became neutral and then filtered out. The solid product was collected, washed with water, followed by cold methanol and then dried up at 50°C. The pure product was afforded by recrystallizing the crude product from the hot ethanol.

3-methoxy-*N*-(6-sulfanyl-9H-purin-2-yl)benzenesulfonamide (4)

Yellow powder; yield 51%; decomp. 280-282°C; υ_{max}/ cm^{-1}(KBr) 3309 (NH); ^{1}H-NMR (DMSO-D_6) δ_H 3.86 (3H, *s*, H22), 6.42 (1H, *s*, H10), 7.12 (1H, *s*, H11), 7.13 (1H, *s*, H2), 7.37 (1H, *dd*, J_{ortho} = 6 Hz, J_{meta} = 2Hz, H18), 7.20 (1H, *t*, J_{ortho} = 7.5 Hz, H17), 12.70 (1H, *br*, *s*, H2); δ_C 55.5 (C22), 108.7 (C20), 111.1 (C18), 114.8 (C17), 118.3 (C16), 129.1 (C4), 138.0 (C15), 153.3 (C2), 159.0 (C19), 160.3 (C9), 168.2 (C5). QTOF-MS m/z calcd for $C_{12}H_{11}N_5O_3S_2[M-H]^+$336.37 found 336.34.

4-methoxy-*N*-(6-sulfanyl-9H-purin-2-yl)benzenesulfonamide (5)

Yellow powder; yield 70%; decomp. 286-289°C; υ_{max}/ cm^{-1}(KBr) 3354 (NH); ^{1}H-NMR (DMSO-D_6) δ_H 3.75 (3H, *s*, H22), 6.90 (1H, *s*, H11), 8.55-8.78 (4H, *m*, H17, H19, H16 and H20), 8.89 (1H, *s*, H), 12.35 (1H, *br*, *s*, H1); δ_C 55.5 (C22), 115.2 (C17 and C19), 128.73 (C16 and C20), 141.5 (C4), 153.4 (C15), 154.2 (C2), 157.5 (C7), 161.1 (C9), 163.0 (C18), 188.9 (C5). QTOF-MS m/z calcd for $C_{12}H_{11}N_5O_3S_2$[M-H]$^+$336.37 found [M-H]$^+$336.3437.

***S*-(2-{[(methylphenyl)sulfanyl]amino}-9H-purin-6-yl)3-methyl-benzenesulfonthioate (6)**

Yellow powder; yield 66%; decomp. 285-287°C; υ_{max}/ cm^{-1} (KBr) 3309 (NH); ^{1}H-NMR (DMSO-D_6) δ_H 2.31 (3H, *s*, H30), 2.43 (3H, *s*, H31), 6.65 (1H, *s*, H13), 7.11 (2H, *d*, J_{ortho} = 6.5 Hz, H20 and H27), 7.20 (1H, *t*, J_{ortho} = 7.5 Hz, H19), 7.39 (1H, *t*, J_{ortho} = 8 Hz, H26), 7.58 (2H, *m*, H22 and H29), 7.64 (2H, *d*, J_{ortho} = 7.5 Hz, H20 and H25), 8.45 (1H, *s*, H2), 12.11 (1H, *br*, *s*, H1); δ_C 21.1 (C30), 21.4 (C31), 123.9 (C25), 125.6 (C29), 126.5 (C22), 127.9 (C26), 128.5 (C19), 129.3 (C27), 130.1 (C18), 136.8 (C20), 137.1 (C17), 138.3 (C28), 140.4 (C21), 140.6 (C24), 153.3 (C2), 153.7 (C5), 155.4 (C7), 160.33 (C9); QTOF-MS m/z calcd for $C_{19}H_{17}N_5O_4S$ [M]$^+$475.56 found 475. 28.

General procedure for synthesis of acetamide-thioguanine compounds

The synthesis of **7-14, 16-18** followed the general procedure for acylation Hu et al., 2010[3]. An amount of 6-thioguanine (0.6 mmol) was mixed with anhydride acetic acid (Ac_2O) (1.2 mL) in 1.2 mL of glaciale acetic acid (GAA). The mixture was refluxed at 135°C for 7.5 hours and the reaction's progress was monitored by TLC using chloroform ($CHCl_3$): acetone (2:2) as a solvent. Once the product was completely formed, the reaction mixture was diluted with 70 mL of ice water and then extracted using 3 x 70 mL of ethyl acetate. The organic phase was collected, washed with 3 x 70 mL of water and then dried with anhydrous magnesium sulfate. This organic phase was then evaporated *in vacuo* to afford the product as a white off powder.

***N*-(6-sulfanyl-9H-purin-2-yl)acetamide (7)**

White powder, yield 87%, m.p. >300°C; ;υ_{max}/ cm^{-1} (KBr) 3109 (NH), 1552 (C=O); ^{1}H-NMR (DMSO-D_6) δ_H 2.21 (3H, *s*, H14), 8.39 (1H, *s*, H2), 11.85 (1H, *br*, *s*, H11), 13.41 (1H, *br*, *s*, H1); δ_C 21.5 (C14), 147.4 (C4), 153.3 (C2), 165.5 (C7), 168.4 (C9), 172.4 (C12), 174.5 (C5). QTOF-MS m/z calcd for $C_7H_5\,K_2N_5OS$ $[M+2K-2H]^+$284.42 found 284.32.

***N*-(6-mercapto-9H-purin-2-yl)butyramide (8)**

White powder, yield 59%, m. p. >300°C; ^{1}H-NMR (DMSO-D_6) δ_H 0.93 (3H, t, H16), 1.63 (3H, m, H15), 2.47 (2H, q, H14), 8.33 (1H, s, H2), 11.82 (1H, br, s, H1); ^{13}C-NMR (DMSO-d_6) 13.9 (C16), 18.4 (C15), 39.4 (C14), 146.8 (C4), 147.5 (C2), 149.8 (C7), 153.3 (C9), 176,8 (C12) QTOF-MS m/z calcd for $C_9H_{14}N_6OS$ $[M+NH_4^{+}+H]^+$256.31 found 256.28.

***N*-(6-mercapto-9H-purin-2-yl)isobutyramide (9)**

The synthesis of **9**followed the procedure for **8** the carboxylic anhydride being used was isobutyricanhydride.White powder, yield 66%, m. p. >300°C; ^{1}H-NMR (DMSO-D_6) δ_H 1.15 (6H, d, H16 and H15), 2.78 (1H, m, H14), 6.45 (1H, br, s, H14), 7.82 (1H, br, s, H11), 8.10 (1H, s, H2), 11.84 (1H, br, s, H1); ^{13}C-NMR (DMSO-d_6) 19.3 (C16 and C15), 35.2 (C14), 126.3 (C4), 145.7 (C2), 153.3 (C9), 178.9 (C12). QTOF-MS m/z calcd for $C_9H_{14}N_6OS$ $[M+NH_4^{+}+H]^+$256.31 found 256.28.

***N*-(6-mercapto-9H-purin-2-yl)pentanamide (10)**

White powder, yield 72%, m. p. >300°C; ^{1}H-NMR (DMSO-D_6) δ_H 1.15 (6H, d, H16 and H15), 2.78 (1H, m, H14), 6.45 (1H, br, s, H14), 7.82 (1H, br, s, H11), 8.10 (1H, s, H2), 11.84 (1H, br, s, H1); ^{13}C-NMR (DMSO-d_6) 14.1 (C17), 22.0 (C16), 28.9 (C15), 36.1 (C14), 147.5 (C2), 153.3 (C9), 177.1 (C12). QTOF-MS m/z calcd for $C_{10}H_{12}N_5OS$ $[M]^+$251.30 found 251.05.

***N*-(6-mercapto-9H-purin-2-yl)-3-methylbutanamide (11)**

White powder, yield 45%, m. p. >300°C; ^{1}H-NMR (DMSO-D$_6$) δ_H 0.89-0.96 (6H, m, H17 and H16), 2.08 (1H, m, H15), 2.37 (2H, dd, H14), 8.36 (1H, s, H2), 11.82 (1H, br, s, H1); ^{13}C-NMR (DMSO-d$_6$) 22.6 (C17 and C16), 25.8 (C15), 45.3 (C14), 147.5 (C2), 176.0 (C5), 176.4 (C12). QTOF-MS m/z calcd for $C_{10}H_{12}N_5OS$ $[M]^+$251.30 found 251.20.

***N*-(6-mercapto-9H-purin-2-yl)hexanamide (12)**

White powder, yield 73%, m. p. >300°C; ^{1}H-NMR (DMSO-D$_6$) δ_H 0.86-0.89 (3H, m, H18), 1.29-1.31 (4H, m, H17 and H16), 1.57 (2H, m, H15), 2.44-2.48 (2H, m, H14), 6.80 (1H, br, s, H10), 7.82 (1H, br, s, H11), 8.09 (1H, s, H2), 12.05 (1H, br, s, H1); ^{13}C-NMR (DMSO-d$_6$) 14.3 (C18), 22.3 (C17), 24.5 (C15), 31.1 (C16), 36.3 (C14), 143.8 (C2), 153.3 (C9), 177.1 (C12)QTOF-MS m/z calcd for $C_{11}H_{15}N_5OS$ $[M+H]^+$266.33 found 266.14.

***N*-(6-mercapto-9H-purin-2-yl)palmitamide (13).**

White powder; yield 70%; decomp. 189-185°C; ^{1}H-NMR (DMSO-D$_6$) δ_H 0.85 (3H, *t*, J = 6.5Hz, H28), 1.23 (24H, *s*, H16-H27), 1.47 (2H, m, H15), 2.18 (2H, *t*, J = 7.5Hz, H14), 6.53 (1H, *s*, H10), 8.13 (1H, *s*, H2), 11.97 (1H, *s*, H11); ^{13}C-NMR (DMSO-D$_6$) δ_C 14.4 (C28), 22.5 (C27), 24.9 (C15 and C16), 31.7 (C26), 34.1 (C14), 39.3- 40.4 (C17-C25), 138.1 (C4), 153.3 (C2), 158.9 (C7), 165.4 (C9), 171.2 (C5), 175.0 (C12). QTOF-MS m/z calcd for $C_{21}H_{38}N_6OS$ $[M+NH_4^++H]^+$422.63 found 422.30.

***N*-(6-mercapto-9H-purin-2-yl)benzamide (M14)**

White powder, yield 49%, m. p. >300°C; ^{1}H-NMR (DMSO-D$_6$) δ_H 6.95 (1H, br, s, H10), 7.49- 7.95 (5H, m. H15-H16), 8.58 (2H, s, H2 and H11), 12.41 (1H, s, H1); ^{13}C-NMR (DMSO-d$_6$) 121.9 (C15 and C19), 129.1 (C16 and C18), 129.7 (C4), 133.4 (C17), 141.5 (C14), 150.1 (C7), 154.3 (C9), 167.8 (C12), 171.9 (5). QTOF-MS m/z calcd for $C_{12}H_9KN_5OS$ $[M+K]^+$310.39 found 310.33.

General procedure for the *N*-alkylation of acetamide-thioguanine

The synthesis of **17 and 20** followed the general procedure for *N*-alkylation Salvatore et al., 2002[4]. Briefly, 138.23 mg of 4 Å Molecular Sieves (MS) was suspended into 3 mL of DMF. Cessium hydroxide monohydrate ($CsOH.H_2O$) (140 µL) was added into the mixture and then stirred up for 10 minutes at the room temperature. The stirring was continued while adding *N*-(6-mercapto-9H-purin-2-yl)acetamide (MH022; 0.5 mmol) into the mixture for 30 minutes at the same temperature. Lastly, cyclopentyl bromide (0.6 mmol) was added into the mixture and the stirring was continued while monitoring the reaction's progress using TLC with $CHCl_3$: acetone (3:1) as a solvent. After the reaction completed, the mixture was diluted with 70 mL of water and then extracted using 3 x 70 mL of EtO-Ac. The organic phase was collected, washed with 3 x 70 mL of water and then dried over anhydrous magnesium sulfate. This organic phase was then evaporated *in vacuo* and purified using PLC to afford the product as a white off powder.

***N*-9-cyclopentyl-6-sulfanyl-9H-purin-2-yl)acetamide (15)**

White powder; yield 86%; m.p 154-156°C; υ_{max}/ cm^{-1} (KBr) 3211 (NH), 1585 (C=O); ^{1}H-NMR (DMSO-D_6) δ_H 1.73 (4H, *m*, H12 and H13), 2.19 (3H, *s*,H19), 2.26 (4H, *m*, H11 and H14), 2.65 (1H. *m*, H10), 8.03 (1H, *s*, H2), 12.85 (1H, *br*, *s*, H16); δ_C 24.0 (C12 and C13), 24.9 (C19), 33.7 (C11 and C14), 56.0 (C10), 128.0 (C4), 142.7 (C2), 149.7 (C7), 152.4 (C9), 161.1 (C17), 169.3 (C5). QTOF-MS m/z calcd for $C_{12}H_{16}N_5OS$ $[M+H]^+$278.35 found 278.13.

***S*-2-(propionamido)-9H-purin-6-yl propanethioate (16)**

White powder, yield 64%, m. p. >300°C; ^{1}H-NMR (DMSO-D_6) δ_H 1.09 (3H, t, H17), 1.19 (3H, t, H19), 2.55 (2H, q, H18), 3.29 (2H, q, H16), 8.61 (1H, s, H2), 11.97 (1H, br, s, H1); ^{13}C-NMR (DMSO-d_6) 8.2 (C19), 9.0 (C17), 29.9 (C18), 30.6 (C16), 133.3 (C4), 140.2 (C2),

144.9 (C5), 148.3 (C7), 153.6 (C9), 172.1 (C14), 178.1 (C11). QTOF-MS m/z calcd for $C_{11}H_{12}N_5O_2S$ $[M-H]^+$278.3108 found 278.1313.

***N*-(6-(3-methylbut-1-en-2-ylthio)-9H-purin-2-yl)isobutyramide (17)**

White powder, yield 72%, m. p. >300°C; ^{1}H-NMR (DMSO-d_6) δ_H 1.11-1.15 (12H, m, H21, H20, H19 and H17), 2.73-2.79 (2H, m, H18 and H16), 8.04 (1H, s, H2), 11.53 (1H, s, H8,), 12.07 (1H, br, s, H1), ; ^{13}C-NMR (DMSO-d_6) 19.3 (C21 and C19), 19.4 (C20 and C17), 35.1 (C18 and C16), 131.9 (C4), 132.3 (C2), 139.7 (C5), 147.6 (C7 and C9), 180.4 (C14) and 180.9 (C11).QTOF-MS m/z calcd for $C_{13}H_{17}N_5O_2S$ $[M\ ]^+$307.37 found 307.10.

***S*-2-(pentanamido)-9H-purin-6-yl pentanethioate (18)**

White powder, yield 43%, m. p. >300°C; ^{1}H-NMR ($CDCl_3$) δ_H 0-66-0.96 (6H, m, H23 and H19), 1.33-1.62 (8H, m, H22, H21, H18 and H17), 2.18-2.40 (4H, m, H20 and H16), 7.32 (1H, br, s, H13), 7.98 (1H, s, H2); ^{13}C-NMR ($CDCl_3$) 14.1 (C23 and C19), 22.7 (C22 and C18), 29.7 (C21 and C17), 31.9 (C20 and C16), 136.5 (C4). QTOF-MS m/z calcd for $C_{15}H_{20}N_5O_2S$ $[M+H]^+$336.42 found 336.34.

General procedure for *S*/*N*-benzylation of acetamide-thioguanine

The synthesis of **19-21** followed the general procedure for S-alkylation Salvatore *et al.*, 2005[5,6]. Briefly, a mixture containing the corresponding alkyl bromide (1.3 mmol, for monoalkylation; 2.6 mmol for dialkylation; 3.9 mmol for triple/quadruple-alkylation), *tetra*-butylammonium iodide (TBAI) (equal to the corresponding number of alkylation) in 3.5 mL of DMF was freshly prepared. In the separate flask, 6-thioguanine (0.6 mmol) was mixed with cessium carbonate (Cs_2CO_3) (equal to the corresponding number of alkylation) in 3.5 mL of dimethylformamide (DMF) and then stirred vigorously for 15 minutes. The first mixture was added into the second mixture and the stirring was continued at room temperature for six

hours. The reaction's progress was monitored by TLC using *n*-hexane: ethyl acetate (1:3) as the mobile phase. After the product formed, the reaction mixture was diluted with 70 mL of water and then extracted using 3 x 70 mL of ethyl acetate. The organic phase was collected, washed with 3 x 70 mL of water and then dried with anhydrous magnesium sulfate. Next, this organic phase was evaporated *in vacuo* and then purified using PTLC with the same mobile phase system used in the monitoring of the reaction's progress.

***N*-(6-(benzylthio)-9H-purin-2-yl)pentanamide (19)**

White powder, yield 30%, m.p., 199-201°C; ^{1}H-NMR ($CDCl_3$) δ_H 0.92-1.01 (3H, m, H23), 1.31-1.33 (4H, m, H22 and H21), 4.59 (2H, s, H11), 7.26-7.48 (5H, m, H17, H16, H15, H14 and H13), 7.90 (1H, s, H2), 8.57 (1H, s, H18); ^{13}C-NMR ($CDCl_3$) 31.8 (C21), 35.2 (C20), 38.3 (C11), 126.8 (C15), 128.11 (C17 and C13), 128.8 (C16 and C14), 138.0 (C4), 168.9 (C19).QTOF-MS m/z calcd for $C_{17}H_{19}N_5OS$ $[M+H]^+$341.13 found 342.32.

6-((naphthalen-1-yl)methylthio)-9-isobutyl-9H-purin-2-amine (20)

White powder, yield 19%, m.p. 143-147°C; ^{1}H-NMR ($CDCl_3$) δ_H 0.94 (6H, s, H26 and H12), 2.19-2.27 (1H, m, H11), 3.86 (2H, s, H10), 5.06 (2H, s, H14), 7.32-7.81 (7H, m, H23-H16), 8.18 (1H, s, H2); ^{13}C-NMR ($CDCl_3$) 20.3 (C26 and C24), 28.9 (C11), 50.8 (C10), 123.8 (C23 and C16), 124.9 (C22 and C21), 127.9 (C18 and C17), 128.5 (C20), 132.5 (C24), 135.4 (C15), 140.5 (C2), 151.0 (C9), 159.0 (C7), 162.0 (C5).QTOF-MS m/z calcd for $C_{20}H_{21}N_5S$ $[M+H]^+$363.15 found 363.17.

***N*-(9-benzyl-6-(benzylthio)-9H-purin-2-yl)pentanamide (21)**

White powder, yield 14%, m.p. 143-147°C;^{1}H-NMR ($CDCl_3$) δ_H 0.93 (3H, m, H31), 1.39-1.42 (4H, m, H30 and H29), 1.69-2.01 (2H, m, H28), 4.59 (2H, s, H18), 5.32 (2H, s, H10), 7.25-7.54 (10H, m, H24-

H20 and H16-H12), 7.82 (1H, br, s, H25), 7.98 (1H, s, H2); ^{13}C-NMR ($CDCl_3$) 13.9 (C31), 22.4 (C30), 29.7 (C29), 30.0 (C28), 33.0 (C18), 47.3 (C10), 127.4 (C14 and C22), 127.8 (C24 and C20), 128.6 (C13, C15, C21 and C23), 129.1 (C12 and C16), 135.1 (C4), 141.9 (C2).). QTOF-MS m/z calcd for $C_{24}H_{25}N_5OS$ $[M]^+$431.18 found 431.15.

References

[1]. Panneerselvam P, Nair RR, Vijayalakshmi G, Subramanian EH, Sridhar SK. Synthesis of Schiff bases of 4-(4-aminophenyl)-morpholine as potential antimicrobial agents. Eur J Med Chem. 2005; 40: 225-229.

[2]. Ramezanian M, Rouge B, La. Tosylation of alcohol. 5,194,651, 16 March 1993, 1993.

[3]. Hu YL, Liu X, Lu M, Ge Q, Liu XB. Synthesis of some biologically actives halogenopurines. J. Kor. Chem. Soc. 2010; 54: 429-436.

[4]. Salvatore RN, Nagle AS, Jung KW. Cessium effect: high chemoselectivity in direct N-alkylation of amines. J Org Chem. 2002; 67: 674-683.

[5]. Salvatore RN, Smith RA, Nischwitz AK, Gavin T. A mild and highly convenient chemoselective alkylation of thiols using Cs_2CO_3-TBAI. Tetrahedron Lett. 2005; 46: 8931-8935.

[6]. Hariono M, Wahab HA, Tan ML, Rosli MM, Razak IA. 9-Benzyl-6-benzylsulfanyl-9H-purin-2-amine. Acta Cryst E. 2014; *70:* o288-o288.
