# Supplementary material for: Thioguanine-based DENV-2 NS2B/NS3 protease inhibitors: Virtual screening, synthesis, biological evaluation and molecular modelling
Source: PLoS One. 2019 Jan 24;14(1):e0210869. doi: 10.1371/journal.pone.0210869 (PMC6345492; doi:10.1371/journal.pone.0210869)
Supplement: S1 Fig — The numbering system is according to the NMR characterisation. (PDF) [file pone.0210869.s002.pdf]

Supporting Information

Thioguanine-based DENV-2 NS2B/NS3 protease inhibitors: Virtual screening, synthesis, biological evaluation and molecular modelling

Maywan Hariono[1,2¶], Sy Bing Choi[1,9&], Ros Fatihah Roslim[1&], Mohamed Sufian Nawi[1,3&], Mei Lan Tan[4], Ezatul Ezleen Kamarulzaman[1], Nornisah Mohamed[1], Rohana Yusof[5], Shatrah Othman[6], Noorsaadah Abd Rahman[6], Rozana Othman[7], Habibah A. Wahab[1,8*]

[1]School of Pharmaceutical Sciences, Universiti Sains Malaysia, Minden, Pulau Pinang, Malaysia

[2]Faculty of Pharmacy, Sanata Dharma University, Maguwoharjo, Sleman, Yogyakarta, Indonesia

[3]Department of Pharmaceutical Chemistry, Kulliyah of Pharmacy, International Islamic University Malaysia, Kuantan, Pahang, Malaysia

[4]Advanced Medical and Dental Institute, Universiti Sains Malaysia, Bertam, Pulau Pinang, Malaysia

[5]Department of Molecular Medicine, Faculty of Medicine, Universiti Malaya, Kuala Lumpur, Malaysia

[6]Department of Chemistry, Faculty of Science, Universiti Malaya, Kuala Lumpur, Malaysia

[7]Department of Pharmacy, Faculty of Medicine, Universiti Malaya, Kuala Lumpur, Malaysia

[8]Malaysian Institute of Pharmaceuticals and Nutraceuticals, Ministry of Science, Technology and Innovation, Halaman Bukit Gambir, Bayan Lepas, Pulau Pinang, Malaysia

[9]School of Data Sciences, Perdana University, Blok B and d1, MAEPS Building, MARDI Complex, Jalan MAEPS Perdana, 43400 Serdang, Selangor

[*]Corresponding Author
E-mail: habibahw@usm.my ; bibwahab@gmail.com

[&]These authors contributed equally to this work

S1 Fig

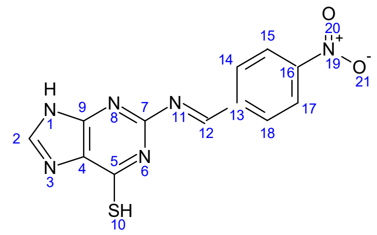

1

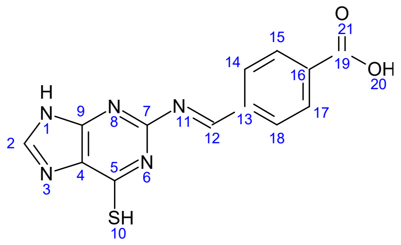

2

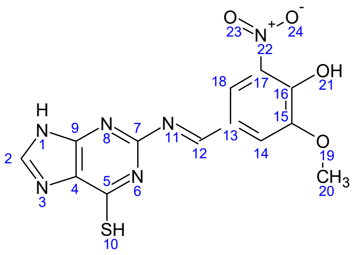

3

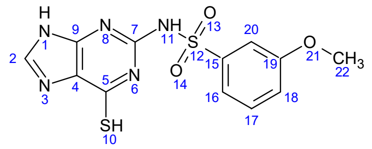

4

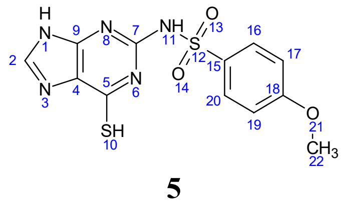

5

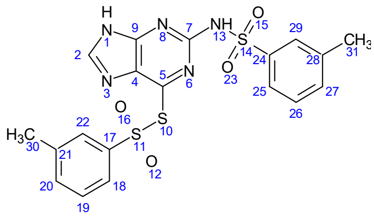

6

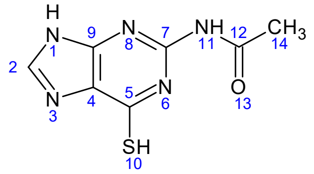

7

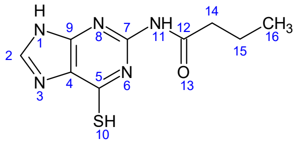

8

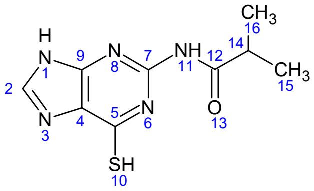

9

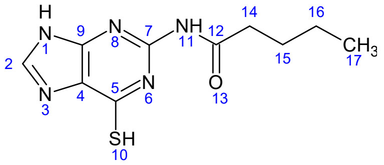

10

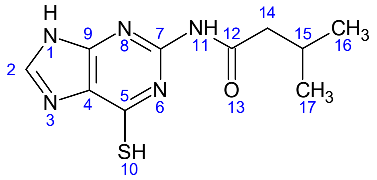

11

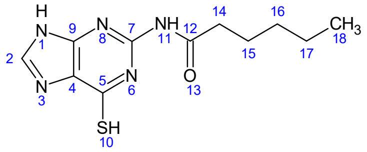

12

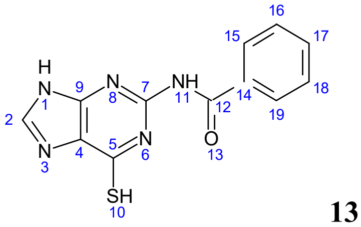

13

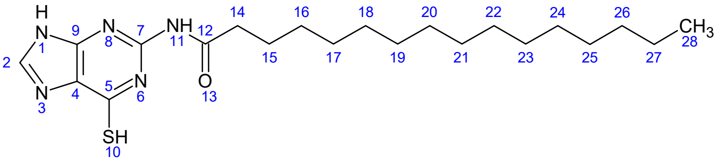

14

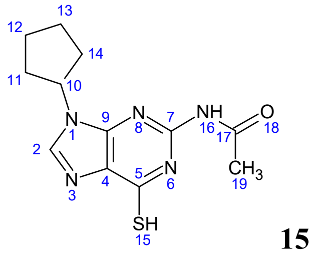

15

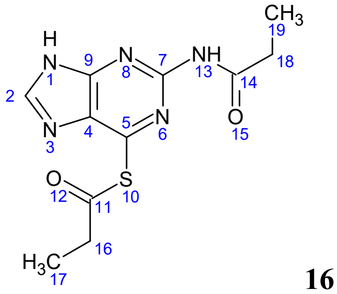

16

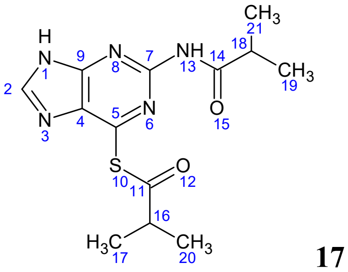

17

S1 Fig. Continue.

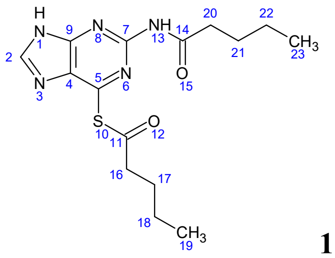

18

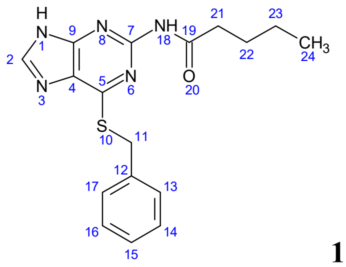

19

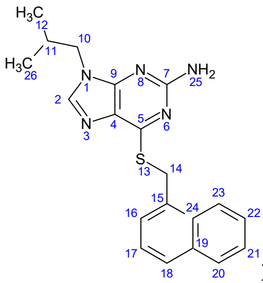

20

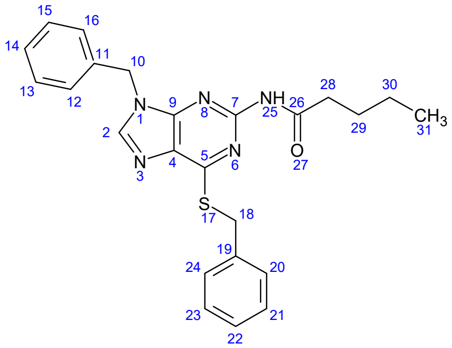

21
