# Supplementary material for: Thioguanine-based DENV-2 NS2B/NS3 protease inhibitors: Virtual screening, synthesis, biological evaluation and molecular modelling
Source: PLoS One. 2019 Jan 24;14(1):e0210869. doi: 10.1371/journal.pone.0210869 (PMC6345492; doi:10.1371/journal.pone.0210869)

Supporting Information

Thioguanine-based DENV-2 NS2B/NS3 protease inhibitors: Virtual screening, synthesis, biological evaluation and molecular modelling

Maywan Hariono[1,2¶], Sy Bing Choi[1,9&], Ros Fatihah Roslim[1&], Mohamed Sufian Nawi[1,3&], Mei Lan Tan[4], Ezatul Ezleen Kamarulzaman[1], Nornisah Mohamed[1], Rohana Yusof[5], Shatrah Othman[6], Noorsaadah Abd Rahman[6], Rozana Othman[7], Habibah A. Wahab[1,8*]

[1]School of Pharmaceutical Sciences, Universiti Sains Malaysia, Minden, Pulau Pinang, Malaysia

[2]Faculty of Pharmacy, Sanata Dharma University, Maguwoharjo, Sleman, Yogyakarta, Indonesia

[3]Department of Pharmaceutical Chemistry, Kulliyah of Pharmacy, International Islamic University Malaysia, Kuantan, Pahang, Malaysia

[4]Advanced Medical and Dental Institute, Universiti Sains Malaysia, Bertam, Pulau Pinang, Malaysia

[5]Department of Molecular Medicine, Faculty of Medicine, Universiti Malaya, Kuala Lumpur, Malaysia

[6]Department of Chemistry, Faculty of Science, Universiti Malaya, Kuala Lumpur, Malaysia

[7]Department of Pharmacy, Faculty of Medicine, Universiti Malaya, Kuala Lumpur, Malaysia

[8]Malaysian Institute of Pharmaceuticals and Nutraceuticals, Ministry of Science, Technology and Innovation, Halaman Bukit Gambir, Bayan Lepas, Pulau Pinang, Malaysia

[9]School of Data Sciences, Perdana University, Blok B and d1, MAEPS Building, MARDI Complex, Jalan MAEPS Perdana, 43400 Serdang, Selangor

[*]Corresponding Author
E-mail: habibahw@usm.my ; bibwahab@gmail.com

[&]These authors contributed equally to this work

2 Fig

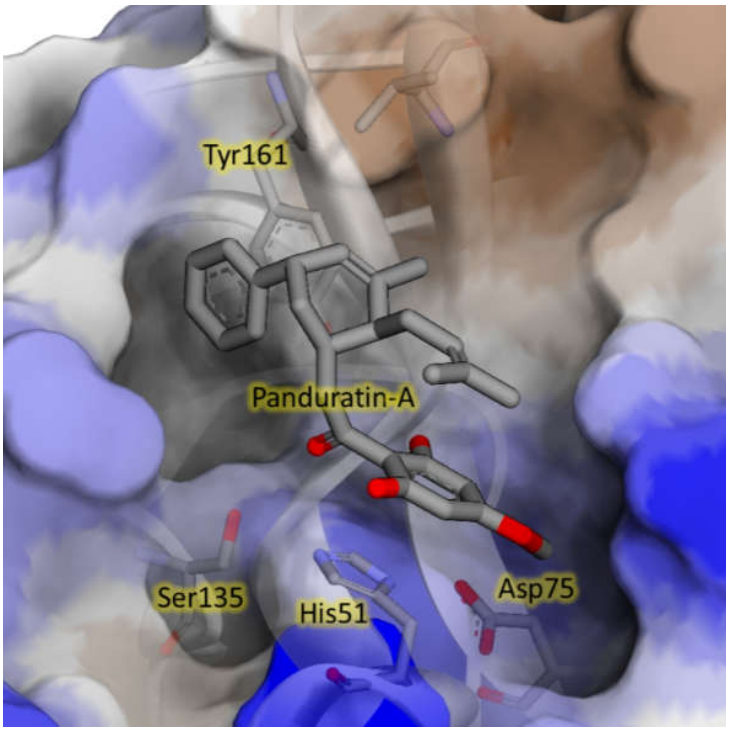

Supplement: S2 Fig — The NS2B-NS3pro is presented in a surface form. The ligands are presented in a stick form in which carbons are grey while oxygens are red. The ligand is docked at the active site of the protease with the hydrophilic moieties surrounded by catalytic site(His51, Asp75 and Ser135) whereas the nonpolar part of the ligand closes nearly to hydrophobic area of the protease wherein phenyl ring posseses H-bond as well as π-π interactions with Tyr161. (PDF) [file pone.0210869.s003.pdf]
