# Supplementary material for: Thioguanine-based DENV-2 NS2B/NS3 protease inhibitors: Virtual screening, synthesis, biological evaluation and molecular modelling
Source: PLoS One. 2019 Jan 24;14(1):e0210869. doi: 10.1371/journal.pone.0210869 (PMC6345492; doi:10.1371/journal.pone.0210869)
Supplement: S1 Table — The NCI compounds are named in two codes and the free energy of binding upon DENV-2 NS2B/NS3pro binding site is expressed in kcal/mol. (PDF) [file pone.0210869.s004.pdf]

Supporting Information

Thioguanine-based DENV-2 NS2B/NS3 protease inhibitors: Virtual screening, synthesis, biological evaluation and molecular modelling

Maywan Hariono[1,2¶], Sy Bing Choi[1,9&], Ros Fatihah Roslim[1&], Mohamed Sufian Nawi[1,3&], Mei Lan Tan[4], Ezatul Ezleen Kamarulzaman[1], Nornisah Mohamed[1], Rohana Yusof[5], Shatrah Othman[6], Noorsaadah Abd Rahman[6], Rozana Othman[7], Habibah A. Wahab[1,8*]

[1]School of Pharmaceutical Sciences, Universiti Sains Malaysia, Minden, Pulau Pinang, Malaysia

[2]Faculty of Pharmacy, Sanata Dharma University, Maguwoharjo, Sleman, Yogyakarta, Indonesia

[3]Department of Pharmaceutical Chemistry, Kulliyah of Pharmacy, International Islamic University Malaysia, Kuantan, Pahang, Malaysia

[4]Advanced Medical and Dental Institute, Universiti Sains Malaysia, Bertam, Pulau Pinang, Malaysia

[5]Department of Molecular Medicine, Faculty of Medicine, Universiti Malaya, Kuala Lumpur, Malaysia

[6]Department of Chemistry, Faculty of Science, Universiti Malaya, Kuala Lumpur, Malaysia

[7]Department of Pharmacy, Faculty of Medicine, Universiti Malaya, Kuala Lumpur, Malaysia

[8]Malaysian Institute of Pharmaceuticals and Nutraceuticals, Ministry of Science, Technology and Innovation, Halaman Bukit Gambir, Bayan Lepas, Pulau Pinang, Malaysia

[9]School of Data Sciences, Perdana University, Blok B and d1, MAEPS Building, MARDI Complex, Jalan MAEPS Perdana, 43400 Serdang, Selangor

[*]Corresponding Author
E-mail: habibahw@usm.my ; bibwahab@gmail.com

[&]These authors contributed equally to this work

S1 Table

Compounds	Structures	ΔG$_{bind}$ (kcal/mol)
NSC11668<br>**(D0126)**	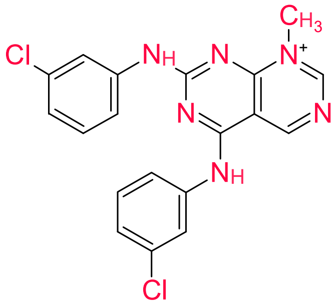	-7.9
NSC13316<br>**(D0152)**	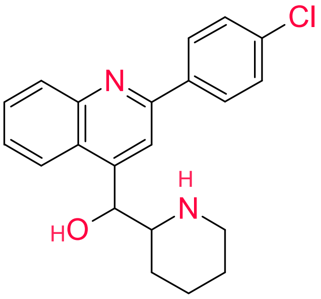	-6.8
NSC13480	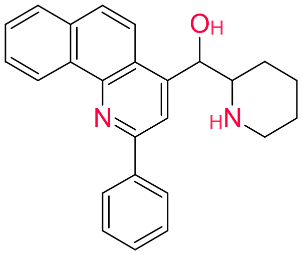	-7.8
NSC17474<br>**(D0227)**	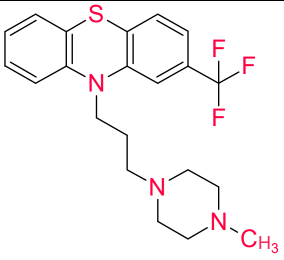	-6.9
NSC19976<br>**(D0265)**	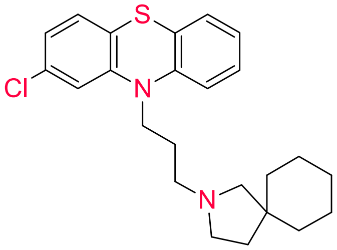	-6.5

NSC37881	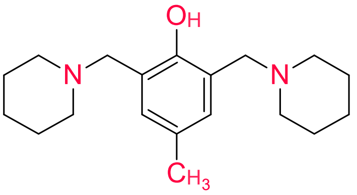	-5.8
NSC54278	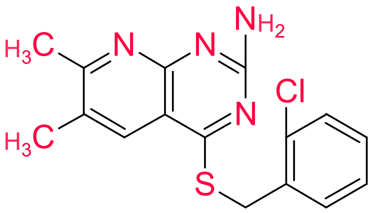	-7.6
NSC54970	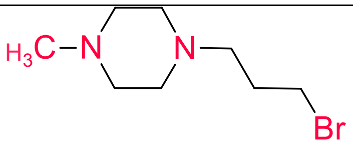	-3.9
NSC56452<br>**(D0685)**	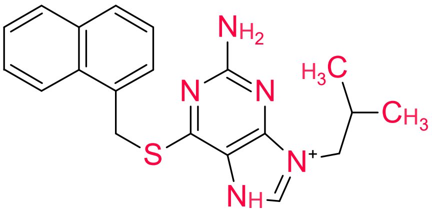	-7.3
NSC59486<br>**(D0713)**	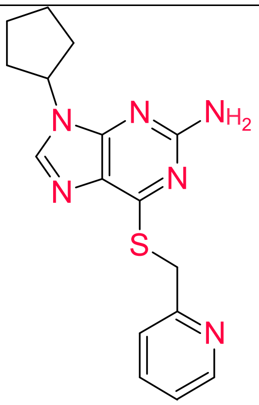	-7.1
NSC116977	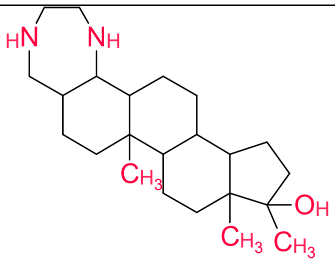	-7.1

NSC99504	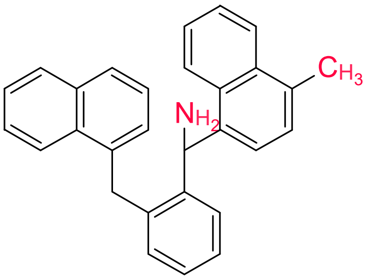	-7.6
NSC118208	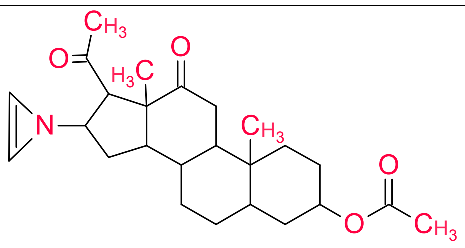	-7.0
NSC122140	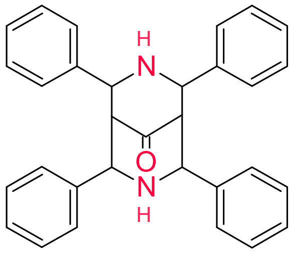	-7.2
NSC47704	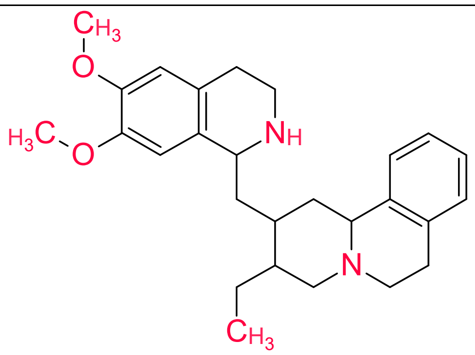	-7.0
NSC34924	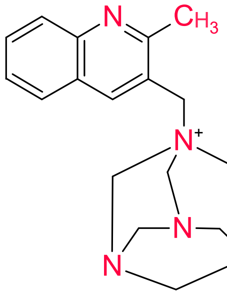	-6.6

NSC170561<br>**(D1498)**	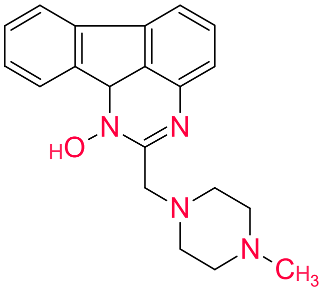	-7.4
NSC211322	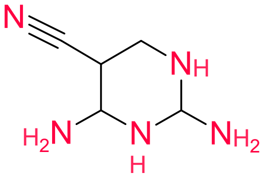	-5.3
NSC48874	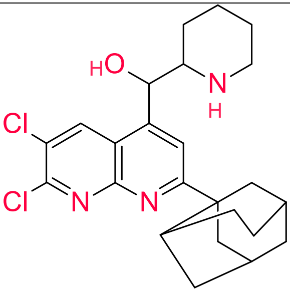	-7.3
NSC350625<br>**(D1804)**	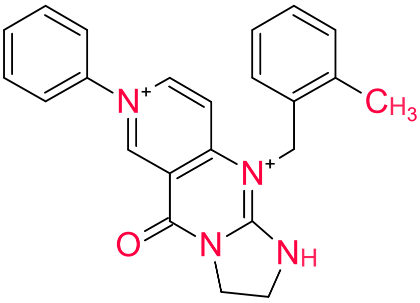	-7.3
NSC371872	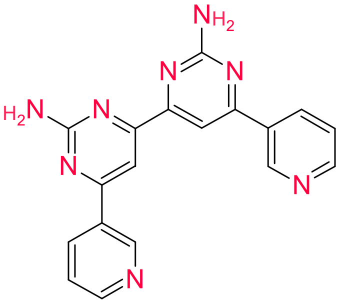	-8.4

NSC371876<br><br>**(D1853)**	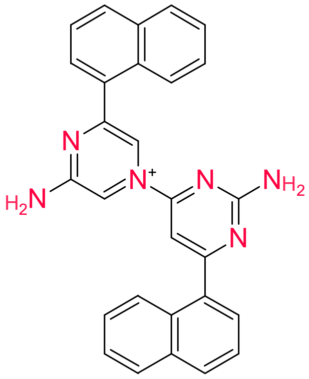	-9.9
NSC371880<br><br>**(D1855)**	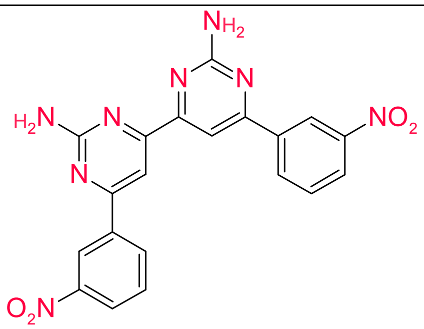	-8.9
NSC372037	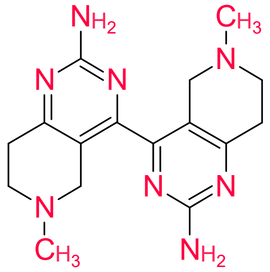	-8.3
